# Synthesis and Thermophysical Properties of Ether‐Functionalized Sulfonium Ionic Liquids as Potential Electrolytes for Electrochemical Applications

**DOI:** 10.1002/cphc.201600882

**Published:** 2016-10-27

**Authors:** Erwan Coadou, Peter Goodrich, Alex R. Neale, Laure Timperman, Christopher Hardacre, Johan Jacquemin, Mérièm Anouti

**Affiliations:** ^1^ The QUILL Research Centre, School of Chemistry and Chemical EngineeringQueen's University of BelfastStranmillis RoadBelfastBT9 5AGUK; ^2^ Laboratoire PCM2EUniversité François RabelaisParc de Grandmont37200ToursFrance; ^3^ School of Chemical Engineering & Analytical ScienceThe University of ManchesterThe MillSackville StreetManchesterM13 9PLUK

**Keywords:** electrochemistry, ether, ionic liquids, physical and transport properties, sulfonium

## Abstract

During this work, a novel series of hydrophobic room temperature ionic liquids (ILs) based on five ether functionalized sulfonium cations bearing the bis{(trifluoromethyl)sulfonyl}imide, [NTf_2_]^−^ anion were synthesized and characterized. Their physicochemical properties, such as density, viscosity and ionic conductivity, electrochemical window, along with thermal properties including phase transition behavior and decomposition temperature, have been measured. All of these ILs showed large liquid range temperature, low viscosity, and good conductivity. Additionally, by combining DFT calculations along with electrochemical characterization it appears that these novel ILs show good electrochemical stability windows, suitable for the potential application as electrolyte materials in electrochemical energy storage devices.

## 1 Introduction

Ionic liquids (ILs) have gained considerable interest over the past few decades due to their numerous attractive properties, such as extremely low vapor pressure, low flammability, high thermal stability and large liquid range. This has led to a number of groups researching IL applications particularly in the fields of catalysis,^1^ and separations.^2^ In comparison with molecular solvents, ILs also possess high ionic conductivity coupled with good electrochemical stability and, therefore, have been touted as new electrolytes for energy devices such as solar cells,^3^ fuel cells,^4^ lithium batteries,^5^ and supercapacitors.^6^

Within the fields of lithium batteries and capacitors, a number of various ILs has been developed with specific physiochemical applications. To date, however, most of these ILs have been based on imidazolium,^7^ tetraalkylammonium,^8^ pyridinium,^9^ and quaternary phosphonium cations.^10^ Despite having a lower viscosity, higher conductivity and lower melting points; ILs based on trialkylsulfonium cations have attracted limited attention compared to their ammonium and phosphonium analogues^11^ with other research groups emphasizing the use of cyclic^12^ and acyclic sulfonium ILs^13^ as electrolytes for energy devices. Recently, we also reported the use of protic and aprotic sulfonium ILs as potential electrolytes for electric double‐layer capacitors (EDLC) devices.^14^

In addition, numerous publications have highlighted the influence of the presence of an ether functionalization on the physiochemical properties of acyclic ammonium‐based^15^ and cyclic ammonium‐based ILs.^16^ Research surrounding the influence of mono‐ether functionalization on the properties of sulfonium‐based ILs has only been studied by Han et al.^17^ and Orita et al.^18^ In this paper, we extended this family of hydrophobic room temperature ILs based on mono‐ether and diether containing alkyl chains appended to sulfonium cations. These ether containing chains are of particular interest for energy storage systems such as supercapacitors and Li batteries due to the possible improved transport properties and good electrochemical stability. Furthermore, the presence of ethereal groups may also offer improved dissociation of the Li^+^ cation and improved transport properties in the bulk solution in Li‐ion batteries, for example. The investigation of viscosity, density, thermal phase behavior, thermal stability, electrochemical stability and ionic conductivity were undertaken. Finally, ILs electrochemical stability results were also analyzed in combination with DFT calculations to further understand the effect of the ether functionalization in the cation on this property.

## 2 Results and Discussion

### 2.1 Synthesis of the Sulfonium ILs

**Figure 1:**
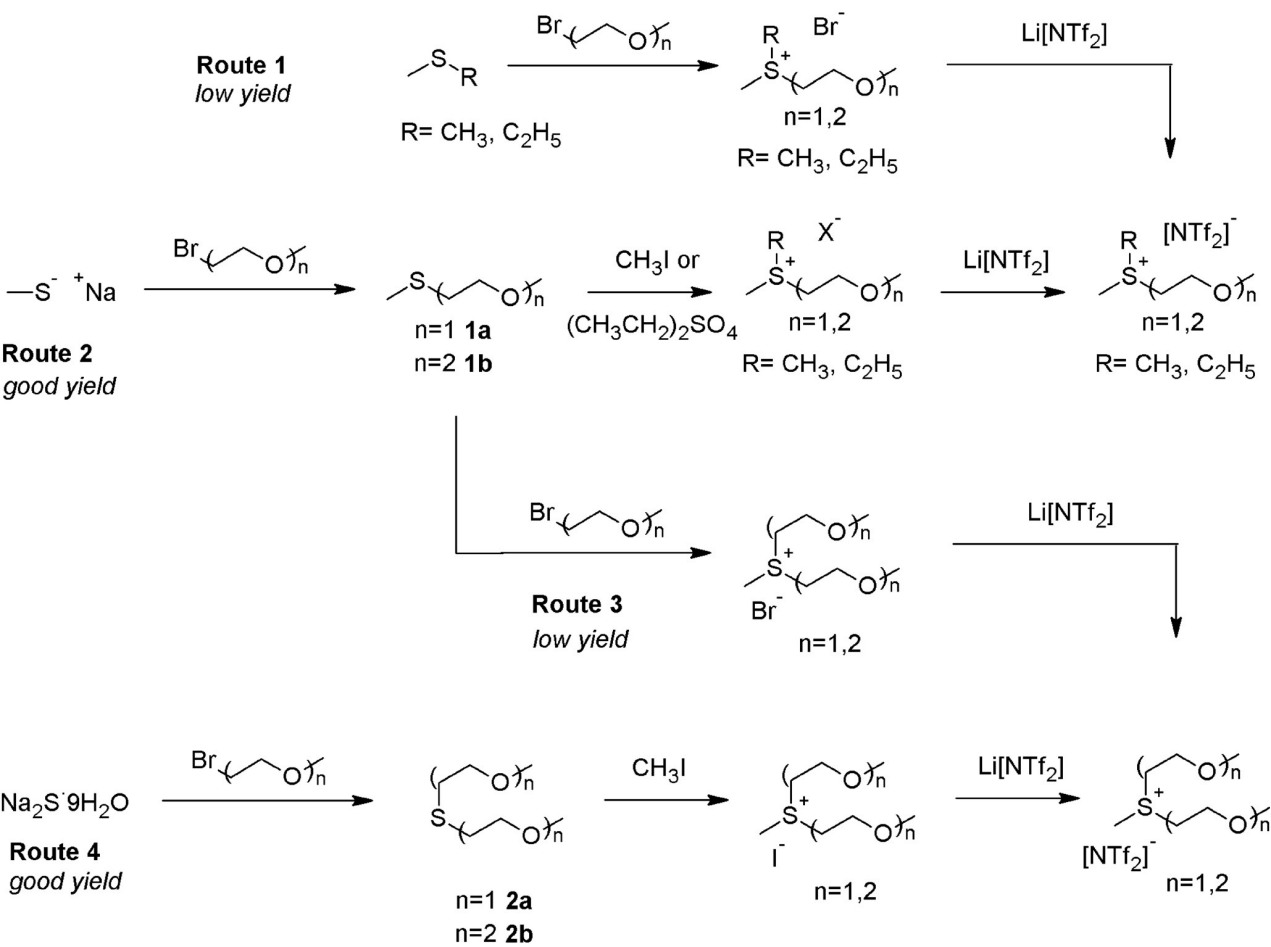
Strategic routes for the synthesis of sulfonium ether ILs.

The general synthesis method of the selected sulfonium based [SR_3_][NTf_2_] ILs is described in the experimental section. The various ether and alkyl substituents R attached to the sulfonium center are listed in Table 1 and the ILs and intermediates (**1 a**, **1 b**, **2 a** and **2 b**) are shown in Figure 1. Several synthetic strategies were undertaken to develop these ILs with the maximum yield and purity. Initial attempts to synthesis these materials using conventional procedures employed for imidazolium‐ or ammonium‐based ILs proved problematic due to the lower alkylation yield observed between the sulfide and the ethers, as shown in Figure 1, route 1.^19^ Therein, low to moderate yields of deeply colored ILs were obtained. Therefore, the proposed strategy was to firstly synthesize the corresponding thioethers **1 a** and **1 b** and, if possible, alkylate at the final step. These thioethers **1 a** and **1 b** were easily synthesized using the conventional Williamson procedure under aqueous conditions.^20^ The corresponding alkylation of the thioethers using either iodomethane or diethylsulfate, via route 2 in Figure 1 also proceeded smoothly to yield the corresponding dialkylether‐sulfonium ILs.

**Table 1:** Abbreviations and alkyl side chains of the sulfonium cations [SR_3_]^+^.

Abbreviation	R^1^	R^2^	R^3^
[S_1,1,G1_][NTf_2_]	CH_3_	CH_3_	CH_2_CH_2_OCH_3_
[S_1,1,G2_][NTf_2_]	CH_3_	CH_3_	(CH_2_CH_2_O)_2_CH_3_
[S_1,2,G1_][NTf_2_]	CH_3_	CH_2_CH_3_	CH_2_CH_2_OCH_3_
[S_1,G1,G1_][NTf_2_]	CH_3_	CH_2_CH_2_OCH_3_	CH_2_CH_2_OCH_3_
[S_1,G2,G2_][NTf_2_]	CH_3_	(CH_2_CH_2_O)_2_CH_3_	(CH_2_CH_2_O)_2_CH_3_

Alkylation of the thioethers **1 a** and **1 b** with other bromoethers to form the alkyldiether sulfonium ILs proved to be more difficult due decreases in nucleophilicity of the sulfur center resulting in lower yields of the desired ILs, see route 3 in Figure 1. Therefore, a different synthetic strategy was adopted to produce alkyldiether‐sulfonium ILs, as shown in Figure 1, route 4. Therein, sodium sulfide was reacted with 2 equivalents of the corresponding bromogylme to form thiodiethers **2 a** and **2 b**. Alkylation of these thioethers with iodomethane proceeded smoothly to furnish the alkyldiether sulfonium iodide which underwent metathesis to form the corresponding [NTf_2_]^−^‐based IL. The metathesis anion exchange reactions were completed in ultrapure water, yielding biphasic systems wherein the [NTf_2_]^−^‐based ILs occupied the bottom, non‐aqueous phase. Undertaking these new synthetic strategies produced colorless ILs in 63–87 % isolated yield (see Experimental Section and Figures S1–S5 of the Supporting Information, SI).

For lab‐scale synthesis of these ether‐functionalised ILs, the commercial availability and good reactivity of 1‐bromo‐2‐(2‐methoxyethoxy)ethane and 2‐bromoethyl methyl ether, make them attractive reagents for relatively straightforward alkylation. However, for larger scale preparation, the bromide containing groups become less feasible. In this context, alternative synthetic strategies wherein alkoxy functionalization is completed using less toxic reagents, (e.g. sulfonate esters, tosylates) have been described in literature and, furthermore, reviewed in depth.^21^

### 2.2 Physical Characterization of the Sulfonium ILs

The thermal properties of selected ILs were characterized by differential scanning calorimetry (DSC) and thermal gravimetric analysis (TGA). Furthermore, the density (*ρ*), dynamic viscosity (*η*) and ionic conductivity (*σ*) were determined as the function of temperature at atmospheric pressure. This characterization data is summarized in Table 2.

**Table 2:** Density, transport and thermal properties of the selected [NTf_2_]^−^‐based sulfonium ILs at atmospheric pressure.

Cation	Mw [g mol^−1^]	*T*_m_ [K]	*T*_f_ [K]	*T*_g_ [K]	*ρ* ^[a]^ [g cm^−3^]	*η* ^[a]^ [mPa s]	*σ* ^[a]^ [mS cm^−1^]	*T*_d_ [K]
[S_1,1,G1_]^+^	401.37	267.54	223.43	189.23	1.5229	46.84	3.94	512
[S_1,1,G2_]^+^	445.42	–	–	194.61	1.4689	33.10	2.85	505
[S_1,2,G1_]^+^	415.39	–	–	215.15	1.4814	30.84	4.93	517
[S_1,G1,G1_]^+^	445.42	–	–	<183^[b]^	1.4638	27.67	3.87	512
[S_1,G2,G2_]^+^	533.52	–	–	194.94	1.3883	35.86	2.34	492

[a] recalculated at 298.15 K using fitting equations (see below). [b] not detectable.

#### 2.2.1 Thermal Properties

The thermal behavior of the studied ILs was investigated by DSC from 183.13 to 373.15 K. As shown in Figure S6 (left) of the SI, most of the ILs only exhibited glass transitions, *T*_g_, with only the [S_1,1,G1_][NTf_2_] functionalized IL showing an observable melting transition, *T*_m_, at 267.54 K and freezing transition, *T*_f_, at 223.43 K. This supercooling phenomenon has been previously well documented for imidazolium‐ and ammonium‐based ILs.^22^

Interestingly, the analogous non‐functionalized [S_1,1,4_][NTf_2_] was observed to have a *T*_m_ between 269.55^23^ and 271.55 K^18^ as measured by two independent research groups. Although we are only comparing one set of ILs, generally, reports in the literature suggest that functionalization of IL alkyl chains with ether groups results in a decrease in melting and increase in liquid range.^24^ Within the ILs studied, increased ether functionalization with either G_1_ (Figure S6 d of the SI) or with G_2_ (Figures S6 b and S6 e of the SI) did result in a significant increase in liquid range. Furthermore, by increasing the alkyl chain length from C_1_ (Figure S6 a of the SI) to C_2_ in (Figure S6 c of the SI) also led to an increase in the liquid range temperature. Therein, the most plausible explanation for the observed lack of *T*_m_ is due to the increased rotational freedom and subsequent reduction in lattice energy as has been described elsewhere.^25^ The *T*_g_ values showed no correlation with regards to the IL functionalization. In addition, as evidenced from the transport studies, the low *T*_g_ values observed do not always lead to low fluidities due to fragility.^26^

To further investigate the thermal properties of the selected sulfonium‐based ILs, TGA analysis of each sample has been carried out. As shown in Figure S6 (right) of the SI, the decomposition temperatures, *T*_d_, of these sulfonium ILs are in the range of 492.15–517.15 K, significantly lower (<150 K) than that observed for analogous ammonium‐based ILs.22b In addition, the short‐chain‐ether functionalized ILs tended to be less thermally stable than their analogous non‐functionalized ILs primarily due to the weakened cation‐anion electrostatic interaction freeing the anions to act as nucleophiles. For example, [S_1,1,G1_][NTf_2_] (Figure S6a of the SI) had a *T*_d_≈38 K lower than the analogous non‐functionalized [S_1,1,4_][NTf_2_] IL.^23^ Increasing the carbon chain from methyl [S_1,1,G1_][NTf_2_] (Figure S6a of the SI) to ethyl [S_1,2,G1_][NTf_2_] (Figure S6 c of the SI) resulted in a slight increase in *T*_d_. The addition of more ether units G_2_ (Figures S6 b and S6 e of the SI) vs. G_1_ (Figures S6 a and S6 d of the SI) resulted in a slight lowering of the decomposition temperature. Despite this lowering of the decomposition temperature, the *T*_d_ values are still significantly higher than those temperatures (373–398 K) encountered in high temperature energy storage devices.^27^ Additionally, as depicted from the Figure S6 of the SI and Table 2, all investigated ILs have a large liquid range temperature, which is higher than 240 K in the case of the [S_1,1,G1_][NTf_2_] and exceeds 300 K for the other ILs. In fact, each IL may be potentially used as electrolytes in low (e.g. 268 K) and/or high (e.g. 473 K) temperatures energy storage devices.

#### 2.2.2 Physicochemical Properties of Selected ILs

The fundamental properties of the sulfonium ILs, including physicochemical quantities of density (*ρ*), viscosity (*η*), conductivity (*σ*), and their ionicity have been investigated as detailed below.

**Figure 2:**
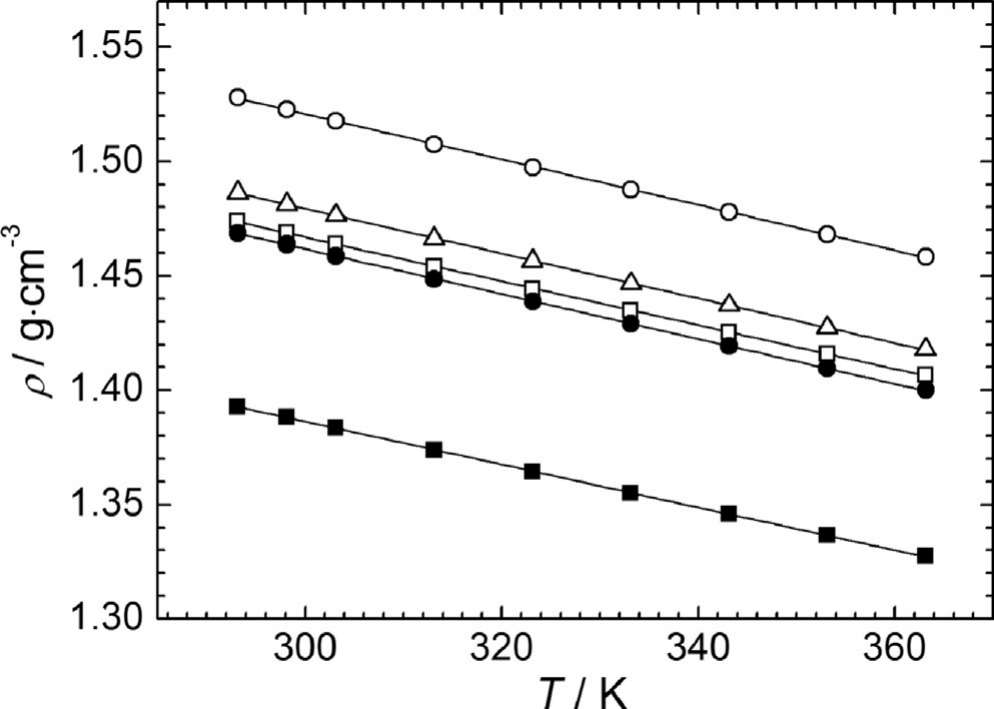
Temperature dependence on the density of [S_1,1,G1_][NTf_2_] (○), [S_1,1,G2_][NTf_2_] (□), [S_1,2,G1_][NTf_2_] (▵), [S_1,G1,G1_][NTf_2_] (•) and [S_1,G2,G2_][NTf_2_] (▪) at 0.1 MPa. The lines represent the linear regression of each density dataset using parameters reported in Table S2 of the SI.

The densities (*ρ*/g cm^−3^) have been measured from 293.15 to 363.15 K at atmospheric pressure for all the investigated ILs (see tabulated data in Table S1 of the SI). As shown in Figure 2, the density of selected sulfonium ILs are between 1.38 and 1.53 g cm^−3^ at 298.15 K, values which are in line with other sulfonium [NTf_2_]^−^‐based ILs previously reported.^23^, ^28^ Furthermore, the density decreases with temperature as expected. Generally, and as shown in Figure 2, over a narrow range of temperatures, the temperature dependence on the density can be expressed as follows:(1)

(1) ρ=a+b·T

where *a*, *b* are the fitting parameters and *T* is the temperature in Kelvin.

Within the ILs studied a strong linear relationship (*R* ^2^>0.9999) with temperature was obtained and the best fitting parameters of Equation (1) are summarized in Table S2 of the SI. Furthermore, as shown in Figure 2, the density of the ILs decreases by increasing the degree of functionalization within the alkyl chain and follows the trend, [S_1,1,G1_]^+^ > [S_1,2,G1_]^+^ > [S_1,1,G2_]^+^ > [S_1,G1,G1_]^+^ > [S_1,G2,G2_]^+^. As the density is governed by the cation‐anion interaction and molecular packing,^29^ which reduces with increasing ether functionalization and/or increase in alkyl chain, this trend mirrors those already reported in the case of the ether functionalized imidazolium‐based ILs.^30^

**Figure 3:**
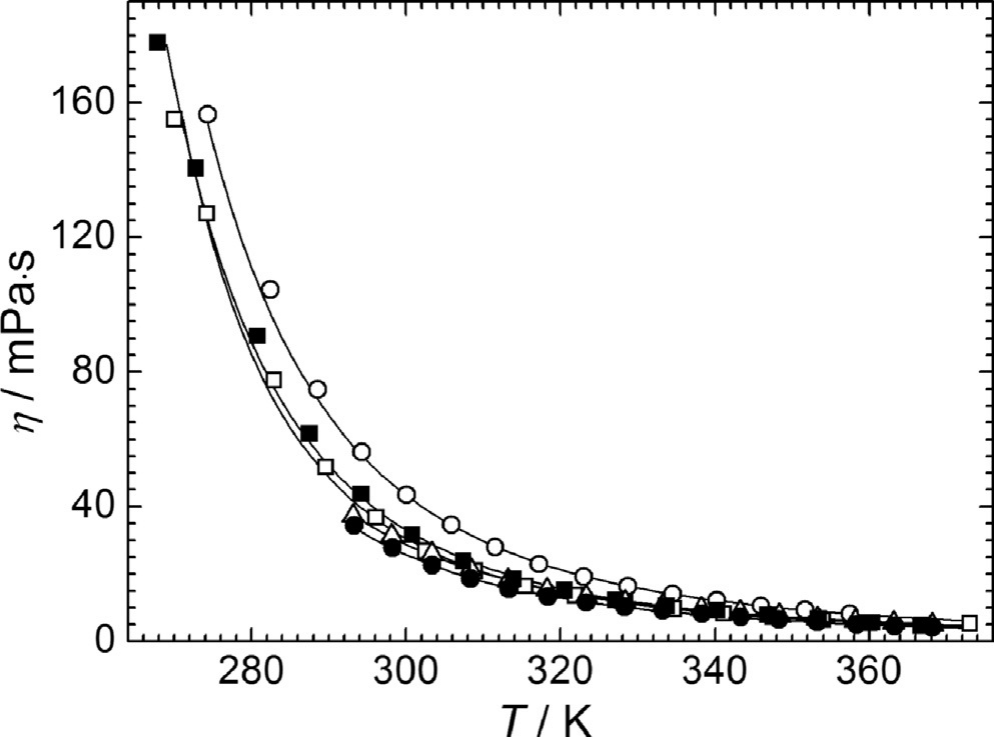
Viscosity of the sulfonium‐based ILs: [S_1,1,G1_][NTf_2_] (○), [S_1,1,G2_][NTf_2_] (□), [S_1,2,G1_][NTf_2_] (▵), [S_1,G1,G1_][NTf_2_] (•) and [S_1,G2,G2_][NTf_2_] (▪) as the function of temperature from 267 K to 370 K at 0.1 MPa. The solid lines correspond to the VTF‐type fitting equations with parameters reported in Table S5 of the SI.

The viscosity (*η*/mPa s) is also an important property for assessing ILs with respect to their use as electrolyte media in energy devices, because of the strong association of the rate of mass transport. The electrolytes in many energy devices are required to operate at near ambient temperature ranges. In other words, any IL used as an electrolyte should have a low viscosity particularly at room temperature. Currently, one of the largest barriers for the application of the ILs as pure electrolytes is their high viscosity in comparison with classical electrolytes for either supercapacitors (55 mS cm^−1^ at 0.6 mPa s for 1 mol dm^−3^ [Et_4_N][BF_4_] in acetonitrile at 298.15 K)^31^ or Li batteries (9.7 mS cm^−1^ at 0.5 mPa s for 1 mol dm^−3^ Li[PF_6_] in ethylene carbonate:dimethylcarbonate blend at 298.15 K).^32^ In this regard, the investigated sulfonium‐based ILs show viscosities ranging from 28 to 53 mPa s at 298.15 K (see Table S3 of the SI). As shown in Figure 3, the viscosity of the ILs decreases with increasing the temperature, as expected. Furthermore, the viscosity seems to be strongly affected by the size and the symmetricity of the cation as well as by the degree of functionalization within the alkyl chain and follows the trend: [S_1,1,G1_]^+^ > [S_1,G2,G2_]^+^ > [S_1,1,G2_]^+^ > [S_1,2,G1_]^+^ > [S_1,G1,G1_]^+^.

To the best of our knowledge, the viscosity of the [S_1,G1,G1_][NTf_2_] IL (27.67 mPa s at 298.15 K) is the lowest of any reported ether functionalized [NTf_2_]^−^ IL, to date. Even the most viscous IL, that is, [S_1,1,G1_][NTf_2_] (46.84 mPa s at 298.15 K) is slightly lower than that of the analogous non‐functionalized sulfonium IL, such as the [S_1,1,4_][NTf_2_] (63.0 mPa s at 298.15 K) previously reported.^18^ This is, however, somewhat higher than the corresponding bis(fluorosulfonyl)imide [FSI]^−^ anion which was reported to have a viscosity of 30.0 mPa s at 298.15 K.^17^ This decrease in viscosity upon the addition of ether units into the alkyl chain is not surprising given the decrease in van der Waals interactions and has also been reported for other ether functionalized ILs.^33^

In addition, all of the ILs showed comparable or significantly lower viscosities than the corresponding mono‐ether containing pyrrolidinium24b (53 mPa s at 298.15 K), piperidinium^34^ (55 mPa s at 298.15 K), linear guandinium^35^ (58 mPa s at 298.15 K) and cyclic guandinium^35^ (46 mPa s at 298.15 K). More surprising, was the similar viscosities observed for the remaining ILs regardless of the degree of ether functionalization. In this regard, negligible differences in viscosity have been reported for di‐ and triether based imidazolium acetate ILs^36^ and the analogous di‐ and trialkyl ammonium acetate based ILs.^37^

**Figure 4:**
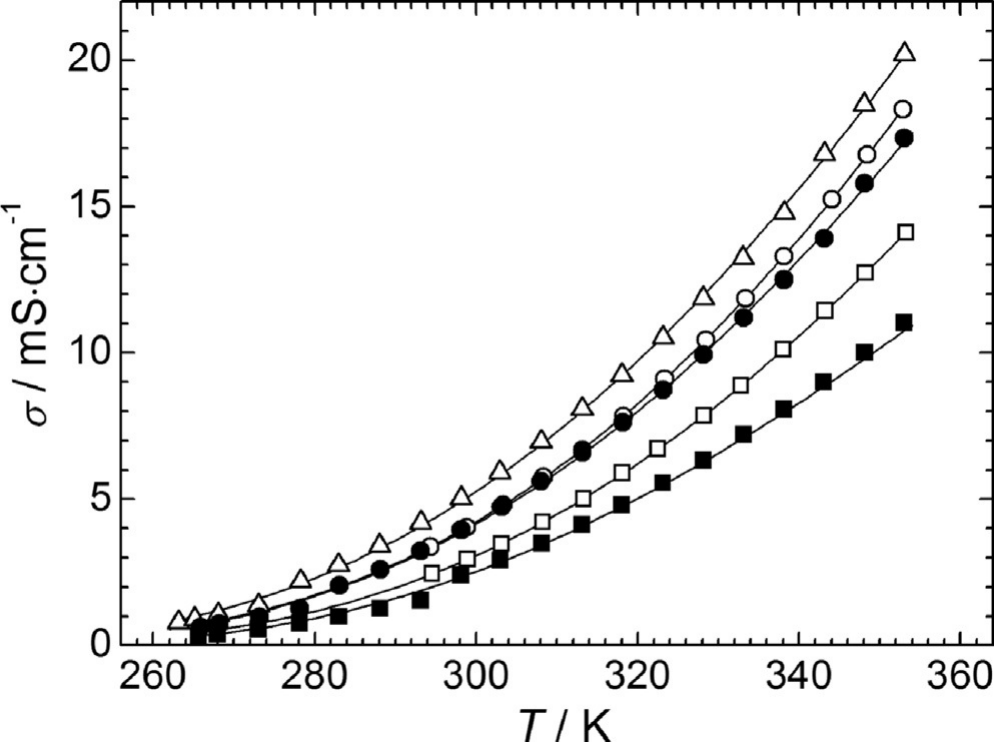
Ionic conductivity of the sulfonium‐based ILs [S_1,1,G1_][NTf_2_] (○), [S_1,1,G2_][NTf_2_] (□), [S_1,2,G1_][NTf_2_] (▵), [S_1,G1,G1_][NTf_2_] (•) and [S_1,G2,G2_][NTf_2_] (▪) as the function of temperature from 263.15 K to 353.15 K at 0.1 MPa. The solid lines correspond to the VTF‐type fitting equations with parameters reported in Table S5 of the SI.

The conductivity of an IL is also of vital importance if it is to be considered as a supporting electrolyte in energy devices. Generally, the ionic conductivity of ILs is mainly governed by their viscosity, formula weight, density and ion size.^38^ As shown in Figure 4, the conductivity of the ILs increases with increasing temperature, as expected. Furthermore, the ionic conductivity of the ILs decreases with the following trend, [S_1,2,G1_]^+^ > [S_1,1,G1_]^+^ > [S_1,G1,G1_]^+^ > [S_1,1,G2_]^+^ > [S_1,G2,G2_]^+^ and covers the range of 2.3–5.0 mS cm^−1^ and of 10.8–20.2 mS cm^−1^ at 298.15 and 353.15 K, respectively (see Table S4 of the SI).

The small difference in ionic conductivity data, even at lower temperatures, suggests that the transport of the ions in these sulfonium ILs is perhaps more dependent on their viscosity. Conversely, for example at 298.15 K, the [S_1,1,G1_][NTf_2_] and [S_1,2,G1_][NTf_2_] ILs show significantly lower ionic conductivities (3.94 and 4.93 mS cm^−1^) compared to the analogous corresponding ILs with the [FSI]^−^ anion (9.5 and 7.6 mS cm^−1^, respectively),^17^ although they have comparable viscosities. This also clearly indicates that an IL containing a common cation and an anion with a lower molecular weight and smaller size is also more favorable for the production of more conductive salts.

The [S_1,G2,G2_][NTf_2_] IL also showed the smallest ionic conductivity (at 298.15 K, *σ*=2.34 mS cm^−1^), despite having a lower viscosity (at 298.15 K, *η*=35.86 mPa s) than the [S_1,1,G1_][NTf_2_] (at 298.15 K, *η*=46.84 mPa s and *σ*=3.94 mS cm^−1^). In this regard, the distinction must be made between these two transport properties. The viscosity measured experimentally is macroscopic in nature, which is clearly related to the cohesive energy of the solution; whereas the conductivity of selected sulfonium‐based ILs seems to be more strongly affected by the structure (volume), interaction (packing) and then the mobility of ions in the solution. One way to investigate the relationship between the viscosity (fluidity) and the conductivity (resistivity) of selected sulfonium‐based ILs is driven by the determination of their ionicity thanks to the utilization of the Walden plot.^26^ Prior to investigation of the effect of the temperature on the ionicity of the studied ILs, each property has to be correlated as the function of the temperature.

Herein, as shown in Table S5 of the SI, all of these ILs deviated slightly from the Arrhenius behavior [Eq. (2)] but could be better described by the Vogel‐Tamman‐Fulcher (VTF) type equation [Eq. (3)]. This is not a surprising result as many other ILs are generally well described by the VTF equation.^39^

(2) $$\eta =\eta_{0}\left[\frac{E_{a}^{\eta }}{\left(RT\right)}\right]\sigma =\sigma_{0}\left[-\frac{E_{a}^{\sigma }}{\left(RT\right)}\right]$$

(3) $$\eta =\eta_{0}\left[\frac{B_{\eta }}{\left(T-T_{0}\right)}\right]\sigma =\sigma_{0}\left[-\frac{B_{\sigma }}{\left(T-T_{0}\right)}\right]$$

where *η*_0_ (mPa s), *σ*_0_ (mS cm^−1^), *E*_a_ (kJ mol^−1^), *B*_*η*/*σ*_ (K), and *T*_0_ (K) are the Arrhenius and VTF fitting constants. The best fitting parameters for the viscosity and conductivity as a function of temperature are reported in the Table S5 of the SI, together with correlation coefficient *R* ^2^ for the fit.

When comparing the ideal glass‐transition temperatures derived from VTF‐type fitting (i.e. *T*_0_ values in the Table S5 of the SI) using the ionic conductivity and viscosity measurements, it can be seen that the corresponding values for each IL show good correlations (i.e. <10 %) except in the case of the [S_1,G2,G2_][NTf_2_] (i.e. ≈22 %). Nevertheless, irrespective of the IL examined, no trend can be observed between *T*_g_ and *T*_0_ values from both VTF‐type fittings. With the exception of [S_1,G2,G2_][NTf_2_], all of the ILs show theoretical *T*_0_ significantly lower than those obtained for the DSC derived *T*_g_ highlighting the fragile nature of these ILs.^40^ The Arrhenius activation energies for both viscosity and conductivity are also depicted in Table S5 of the SI, and are calculated between the temperature range 293.15 and 353.15 K. As expected, for each IL the activation energy for conductivity is lower than that for viscosity due to the fractional Walden rule.^41^

Unlike the Arrhenius equation, all VTF equations seem to correlate accurately each property over a wider temperature range allowing better predictions for both the limiting viscosity (*η*_0_) and limiting conductivity (*σ*_0_), see Table S5 of the SI. This correlation allows better determination of the Walden ionicity of the selected ILs as a function structure and temperature. Prior to constructing the Walden plot, the molar conductivity (Λ_m_/S cm^2^ mol^−1^) data must be calculated within the same temperature range for each IL according to Equation (4):

(4) $$\Lambda_{m}=\sigma \cdot \frac{Mw}{\rho }$$

where, *σ* (S cm^−1^), *Mw* (g mol^−1^) and *ρ* (g cm^−3^) are the ionic conductivity, the molecular weight and the density of the selected ILs.

**Figure 5:**
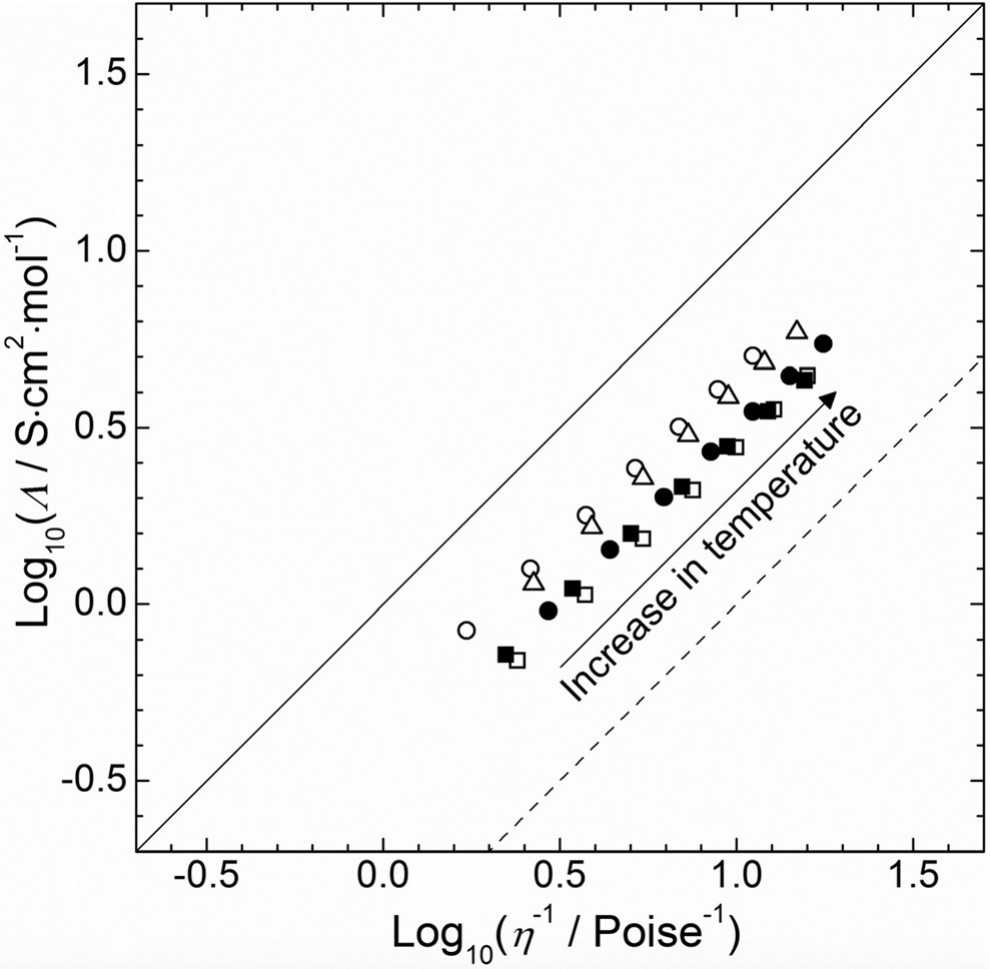
Walden plot of temperature‐dependent conductivities and viscosities for selected sulfonium‐based ILs: [S_1,1,G1_][NTf_2_] (○), [S_1,1,G2_][NTf_2_] (□), [S_1,2,G1_][NTf_2_] (▵), [S_1,G1,G1_][NTf_2_] (•) and [S_1,G2,G2_][NTf_2_] (▪). The solid line corresponds to the Walden plot ideal line corresponding to aqueous KCl solution. The dashed line corresponds to an ionicity of 10 %.

The Walden plot (i.e. the variation of log_10_(Λ_m_/S cm^2^ mol^−1^) vs. log_10_(*η* ^−1^/P^−1^)) for the five neat ILs within a temperature range of 293.15 to 353.15 K is presented in Figure 5. The specific points on the graph were calculated using the correlation parameters of the fitting equations for density [Eq. (1) with parameters reported in Table S2 of the SI], conductivity, and viscosity [Eq. (3) with parameters reported in Table S5 of the SI] from 293.15 to 353.15 K within steps of 10 K. The solid line in Figure 5 represents a so‐called ideal KCl line, that is, the ideal Walden behavior of a 0.01 mol dm^−3^ aqueous solution of KCl, a strong electrolyte, where the constituent ions are known to be fully dissociated and equally mobile in solution.^42^ As shown in Figure 5, a deviation below this ideal line is observed for all investigated sulfonium‐based ILs, which may indicate, in each case, that not all ionic species are available for the conduction of charge and their apparent electrolytic conductivity is lower than may be expected for a given viscosity. In other words, this may be related to a slight tendency for the formation of ion pairs in each IL. This is not a surprising result as such behavior has been reported for several ILs.^43^ Furthermore, as reported in Table S6 of the SI, by applying the “*classical*” Walden rule, it appears clearly that the corresponding Walden product, *W*=Λ_m_⋅*η* is temperature dependent. Again, this behavior is already well described in the literature for a large range of ILs, and according to Schreiner et al.,^43^ a better illustration of the conductivity‐viscosity relationship could be achieved by using a fractional Walden rule as follows:(5)

(5) $$W^{{}^{'}}=\Lambda_{m}\cdot \eta^{\alpha }↔\;{log}_{10}\left(\Lambda_{m}\right)={log}_{10}\left(W^{{}^{'}}\right)+\alpha \cdot {log}_{10}\left(\frac{1}{\eta }\right)$$

where *α* and *W*′ are an additional exponent fitting parameter and the “Walden product” of the fractional Walden rule.

For all investigated ILs, excellent correlations have been achieved by applying the fractional Walden rule [Eq. (5)] with slopes (i.e. *α*) and intercepts (i.e. log(*W*′/S cm^2^ mol^−1^)) ranging from (0.91 to 0.99) and from (−0.29 to −0.53), respectively. The linear fitting parameters for each IL are shown in Table S7 in the SI.

**Figure 6:**
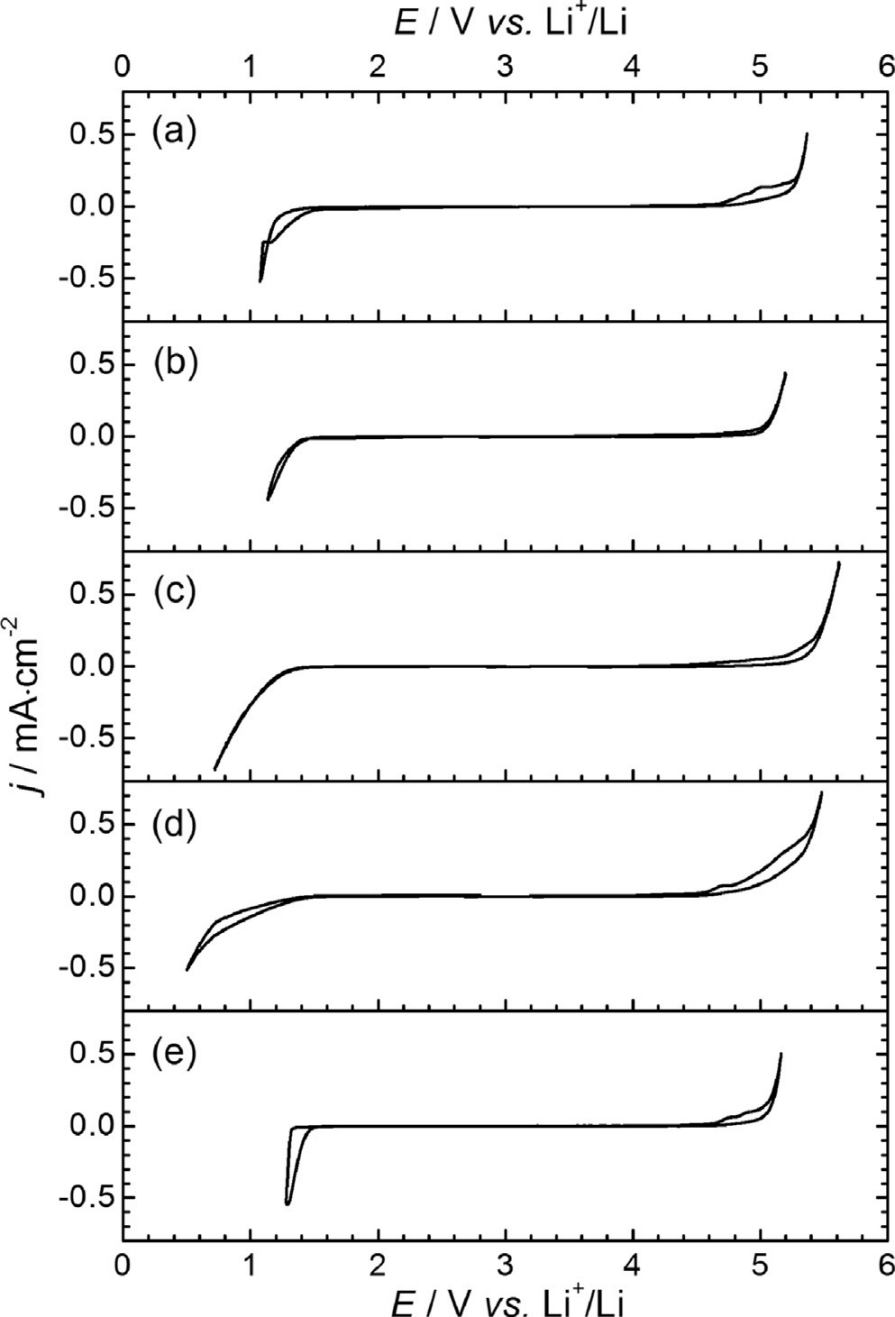
Electrochemical windows of [S_1,1,G1_][NTf_2_] (a), [S_1,1,G2_][NTf_2_] (b), [S_1,2,G1_][NTf_2_] (c), [S_1,G1,G1_][NTf_2_] (d) and [S_1,G2,G2_][NTf_2_] (e).

Based on parameters reported in Table S7, the ionicity of all the five ILs range between 30 % and 50 %. Furthermore, the ionicity seems to decrease with increasing the degree of asymmetry and functionalization within the alkyl chain and follows the trend: [S_1,1,G1_]^+^ (50 %) > [S_1,2,G1_]^+^ (45 %) ≫ [S_1,G2,G2_]^+^ (35 %) ≈ [S_1,G1,G1_]^+^ (34 %) ≥ [S_1,1,G2_]^+^ (29 %). In other words, an increase of the degree of asymmetry and functionalization on the sulfonium cation seems to induce an increase of ion‐pairing in solution. Although, the ionicity values are lower than those determined for the 1‐ethyl‐3‐methylimidazolium tetrafluoroborate ([C_2_mim][BF_4_], 96 %)^43^, the 1‐butyl‐3‐methylimidazolium tetrafluoroborate ([C_4_mim][BF_4_], 64 %)^42^ or the 1‐butyl‐1‐methylpyrrolidium bis{(trifluoromethyl)sulfonyl}imide ([C_4_mpyrr][NTf_2_], 72 %)^44^ the ILs reported are similar than those calculated for the [S_1,*x*,*y*_][NTf_2_] series with *x*=1 to 3 and *y*=2 to 5 (38–56 %).^28^ In other words, all investigated ILs could be considered as “good” ILs. The threshold between the classification of “good” and “poor” ILs, originally proposed by Angell^45^ is represented by the dashed diagonal line in Figure 6 and corresponds to an ionicity of 10 %. ILs which lie below this line have a strong tendency for ion‐pair formation.

#### 2.2.3 Electrochemical Window

One of the reasons for the recent growing interest in ILs is the wide electrochemical windows, which may allow them to be used as solvent‐free supporting electrolytes in high‐energy density devices, including LiS batteries and electrochemical capacitors.^46^ Therefore, of particular interest is the relationship between the cationic structures of the ILs and their electrochemical windows. Cyclic voltammograms (CVs) have been measured (Figure 6) for all investigated ILs using a 3‐electrode cell inside an Ar filled glove box, with a 0.3 cm diameter glassy carbon disk working electrode, a fresh lithium strip as reference electrode and a platinum wire as counter electrode. A small quantity of Li[NTf_2_] salt (i.e. 20 mg of Li salt per mL of IL) has been added to the IL to be used as an internal reference.

The reduction (*E*_red_) and oxidation (*E*_ox_) limiting potentials of the ILs were then determined by a graphical method at the potential value where tangent lines to the main reduction/oxidation walls and the “flat” electroactivity domains cross. These values are presented in the Table 3. The reduction limiting potentials suggest that both the number and the length of the ether chains attached on the sulfonium cation have a negligible effect on the reduction potential of the ILs. However, such values seem to be lower than those reported for [S_1,1,4_][NTf_2_] (0.9 V vs. Li^+^/Li).^28^ This difference may be explained by comparing the LUMO energy of selected cations (average close to −4.39 eV) vs. [S_1,1,4_]^+^ (−4.60 eV) as shown in Table S8 of the SI.

**Table 3:** Oxidation (*E*_ox_) and reduction (*E*_red_) limiting potentials and electrochemical windows (Δ*E*) of selected [NTf_2_]^‐^‐based sulfonium ILs.

Cation	*E*_ox_ [V vs. Li^+^/Li]	*E*_red_ [V vs. Li^+^/Li]	Δ*E* [V]
[S_1,1,G1_]^+^	5.09	1.34	3.75
[S_1,1,G2_]^+^	4.94	1.37	3.57
[S_1,2,G1_]^+^	5.11	1.24	3.87
[S_1,G1,G1_]^+^	4.75	1.36	3.39
[S_1,G2,G2_]^+^	4.69	1.36	3.33

Furthermore, a comparison between the reduction potential of [S_1,1,G1_][NTf_2_] (e.g. 1.34 V vs. Li^+^/Li and LUMO=−4.58 eV) and [S_1,2,G1_][NTf_2_] (e.g. 1.24 V vs. Li^+^/Li and LUMO=−4.60 eV) tends to indicate that the electrochemical stability of this IL series increases by increasing the alkyl chain length, as already reported in the case of several other ILs.^47^

On the other hand, the data reported in Table 3 shows that the length of the ether chains has an important effect on the oxidation potential of the ILs even if they are all based on the same anion. For example, a comparison of these data between [S_1,1,G1_][NTf_2_], [S_1,2,G1_][NTf_2_] and [S_1,1,G2_][NTf_2_] clearly indicates that the oxidation potential decreases by increasing the degree of functionalization of the IL. A similar trend is also observed by comparing the data for [S_1,G1,G1_][NTf_2_] versus [S_1,G2,G2_][NTf_2_] and for [S_1,1,G2_][NTf_2_] versus [S_1,G2,G2_][NTf_2_]. As the reduction limiting potentials do not seem to be affected by the degree of functionalization, contrary to the oxidation limiting potentials, it can be concluded that the degree of functionalization of the cation does not impact the reduction of the ILs (which is quite surprising) while it does influence the environment (interaction) of the [NTf_2_]^−^ anion and thus its oxidative stability. The impact of the degree of functionalization on the oxidation potential of the cations was then further examined by comparing the HOMO energy of selected cations determined from DFT calculations as shown in Table S8 of the SI. From these data, it can be observed that the HOMO energy is always localized on the oxygen atom furthest from the sulfonium centre and seems to decrease by increasing the degree of functionalization. For example, HOMO energies close to −11.20; −9.97; −9.55 eV were observed for the [S_1,1,G1_][NTf_2_], [S_1,1,G2_][NTf_2_], [S_1,G2,G2_][NTf_2_], respectively. However, such energies are much lower (i.e. more stable toward the oxidation) than those determined for each conformation of the [NTf_2_]^−^ anion (e.g. −4.19; −3.97 and −3.89 eV; see Table S8 of the SI), as expected. In other words, all DFT calculations done during this work (individual ions and ion pair) seem to reveal that only the anion may participate, directly, to the oxidation of each IL. However, stronger cation‐anion interaction may be expected for ILs presenting a stronger tendency to form ion pairs in solution. Interestingly, by comparing results of the ionicity and oxidation limiting potentials, it appears than both properties seem to be correlated, as ILs presenting lower ionicity levels, such as [S_1,G1,G1_][NTf_2_], [S_1,1,G2_][NTf_2_] and [S_1,G2,G2_][NTf_2_], have also lower oxidation potentials. This may be associated to the fact that both the cation and anion have less mobility/configuration freedom than those in [S_1,1,G1_][NTf_2_] or [S_1,2,G1_][NTf_2_]. On the one hand, strong cation‐anion interaction may indicate the possibility for the cation to participate to the oxidation process as claimed by some authors,^48^ on the other hand, a low degree of freedom may also influence the probability of finding various anion and cation conformations in solution. Interestingly, DFT calculations of the ion pair [S_1,G1,G1_][NTf_2_] suggest that the conformation *cis*‐[NTf_2_]^−^ surrounding the cation is the most stable, for example (see Table S8 of the SI). However, in the case of the single anion DFT calculations, this conformation is not the most stable and led to an increase of the anion HOMO energy close to +0.22 eV versus the *trans*‐conformer. In both cases, the degree of functionalization of the cation seems to impact, strongly, on the oxidation potential of the ILs. This may be driven by a change on the cation‐anion interactions inducing a radical change of the anion conformer ratio (*cis* vs. *trans*) involving a decrease of the oxidation potential. This hypothesis could also explain why the electrochemical windows of the selected ILs, which range from 3.3 to 3.9 V, are lower than those reported for the [S_1,1,*x*_][NTf_2_] series with *x*=2 to 5 (i.e. from 3.8 to 4.2 V).^28^

## 3 Conclusions

A novel family of cation‐functionalized sulfonium ILs has been synthesized and characterized. Different synthesis routes have been investigated to be able to make targeted ILs in large scale within a good yield (63–87 % yield). Firstly, thioethers were made by following the conventional Williamson procedure under aqueous conditions. Then, dialkyl‐ether‐sulfonium‐based ILs can be easily synthesized with a good yield by the alkylation of the thioethers using either iodomethane or diethylsulfate. Similarly, based on our investigations, alkyl‐diether‐sulfonium‐based ILs can only be obtained with a good yield by the alkylation of the thiodiethers using iodomethane. These thiodiethers were obtained by reacting sodium sulfide with 2 equivalents of the corresponding bromogylme. In each case, anion exchange was achieved by a simple metathesis reaction with Li[NTf_2_] salt.

These ILs exhibit good thermal stability, low melting points, and good transport properties. As expected, the structure of the cation affects strongly all investigated properties. Both the density and conductivity decrease by increasing the degree of functionalization, while more complex relationships were observed in the case of the viscosity. Furthermore, according to the Walden rule, all investigated ILs can be classified as “good” ionic liquids with ionicity ranging from 30 % to 50 %. The ionicity seems to be strongly affected by the degree of asymmetry and of functionalization on the cation structure. The [S_1,1,G1_][NTf_2_] has the highest ionicity (50 %), followed by the [S_1,2,G1_][NTf_2_] (45 %), while lower values (29–35 %) have been obtained for [S_1,G1,G1_][NTf_2_], [S_1,1,G2_][NTf_2_] and [S_1,G2,G2_][NTf_2_]. Interestingly, a similar trend was observed for the electrochemical windows of these ILs with a strong impact of the degree of functionalization of the cation on this property. This impact may be related to strong interactions between functionalized cation and the [NTf_2_]^−^ anion leading to a slight decrease of the oxidation limiting potential of these ILs. However, these functionalized sulfonium ILs show quite large electrochemical windows higher than 3.3 V. In other words, the results obtained during this work indicate that these ILs could be applied in electrochemical energy storage devices. In particular, the application of this class of ether‐functionalized sulfonium [NTf_2_]^−^‐based ILs in electrochemical double layer capacitors is currently being investigated.

## Experimental Section

### Materials Used During the ILs Synthesis

Dimethylsulfide (98 %), sodium thiomethoxide (21 wt % in H_2_O), sodium sulfide nonahydrate (98 %) and 1‐bromo‐2‐(2‐methoxyethoxy)ethane (95 %) were purchased from Sigma‐Alrich. 2‐Bromoethyl methyl ether (95 %) was purchased from Fluorochem. Lithium bis{(trifluoromethyl)sulfonyl}imide (Li[NTf_2_]) was purchased from 3 m. All solvents purchased from MACRON were of HPLC grade and used as received.

### Methods Used During the ILs Characterization

Prior to any physiochemical, thermophysical and electrochemical measurement, all the ILs were dried under high vacuum (2×10^−3^ mbar) for 48 h at 343.15 K. The water content in ILs was analyzed by means of a coulometric Karl‐Fischer titration using an 899 Coulometer (Metrohm) with an accuracy better than 10 ppm.

Density measurements were performed using a DM40 (Mettler Toledo) oscillating tube densitometer in the range of 293.15–363.15 K (±0.01 K) within an accuracy close to ±10^−4^ g cm^−3^. Prior to any measurements, the instrument was cleaned with acetone and dried with dehumidified air.

The viscosity of the ILs was measured using a Bohlin Gemini Rotonetic Drive 2 cone and plate rheometer from 267 to 370 K (±0.01 K) at atmospheric pressure. The viscosity standard (ASTM Oil Standard S600 of CANNON, 1053 mPa s at 298.15 K) and ultra‐pure water were used to calibrate the viscometer. Based on these measurements, the accuracy of reported viscosity measurements is close to ±1 %.

Dynamic thermogravimetric analysis (TGA) of each IL was determined using a TGA Q5000 (TA Instruments) under nitrogen flow with a heating rate and terminal temperature set at 5 K min^−1^ and 773.15 K, respectively. The decomposition temperature onset, *T*_d_, is taken when the samples had lost 5 % of their initial masses. Reported thermal properties are given with accuracy close to ±1 K.

Thermal phase transitions of each IL were recorded using differential scanning calorimetry (DSC) traces on a DSC Q2000 (TA Instruments). Hermetically sealed aluminum pans containing the respective IL sample were prepared inside the Ar‐filled glove box for DSC analysis. A sample of average weight of ≈5 mg was hermetically sealed in an aluminum pan, and then heated and cooled at a rate of 5 K min^−1^ from 183.15 to 273.15 K under a flow of nitrogen. The glass transition temperature (*T*_g_, onset of the heat capacity change), crystallization temperature (*T*_c_, onset of the exothermic peak), and melting point (*T*_m_, onset of the endothermic peak) were recorded on the first or second heating scans with accuracy close to ±0.25 K.

Conductivity measurements were performed using a sensION+ EC71 benchtop meter with a 3‐pole platinum sensION+ 5070 conductivity probe with an in‐built Pt1000 temperature probe (Hach Lange). The conductivity probe was calibrated using aqueous KCl standard conductivity solutions (147 μS cm^−1^, 1413 μS cm^−1^, and 12.88 mS cm^−1^ at 298.15 K). The immersion and sealing of the conductivity probe in the liquid sample was carried out in an Ar‐filled glovebox. The conductivity probe (disconnected from the meter) was immersed in the liquid sample inside a glass sample tube with a small magnetic stirrer. The sample was sealed using an O‐ring seal and parafilm. The conductivity of the sample was then recorded with stirring as a function of temperature (using the temperature reading built into the conductivity probe). The temperature of the sample was varied from 263.15 to 353.15 K using a small oil bath and a hot‐plate with a thermocouple control. The temperature and conductivity of the sample was recorded when the values were stable for about1 min with accuracies close to 0.05 K and 1 %, respectively.

All electrochemical measurements were performed using a Versatile multichannel potentiostat (VMP 3, Biologic S.A.) inside an Ar‐filled glovebox. The measurements have been conducted at a scan rate of 2 mV s^−1^ in a 3‐electrode cell using a 0.3 cm diameter glassy carbon as the working electrode, a fresh lithium metal strip as the reference, and a platinum wire as the counter electrode. The electrolyte consisted of ca. 1 mL of pure IL in which a small quantity of Li[NTf_2_] (approx. 20 mg) was dissolved to introduce a Li^+^/Li reference system.

HOMO and LUMO energies of each species have been determined by using the Turbomole 7.0 program package.^49^ Prior to visualization of these orbitals using TmoleX (version 4.1.1), the structure of each ion involved was optimized, with a convergence criterion of 10^−8^ Hartree in the gas phase, by using DFT calculations combining the Resolution of Identity (RI) approximation^50^ within the Turbomole 7.0 program package utilizing the B3LYP functional with the def‐TZVP basis set.^51^ Each resultant optimized structure was then used as an input for the generation of the conformers of each species using the COSMOconfX program (version 4.0). The orbitals of each conformer were then determined using single point energy calculations (DFT/B3LYP/def‐TZVP + RI approximation) within Turbomole.

### General Synthesis of the Sulfonium‐based ILs

**Synthesis of the Thioethers 1 a and 1 b**: To a flask containing a 21 wt % aqueous solution of sodium thiomethoxide (182.942 g, 0.548 mol, 1.1 equiv.) was slowly added, over 2 h, the corresponding bromoether (0.498 mol, 1 equiv.) The reaction mixture was covered by an aluminum foil and immersed in an ice bath. After complete addition of the bromoether, the mixture was stirred and allowed to return slowly to room temperature for approximately 15 h. To this was added 50 mL of diethyl ether to dissolve and extract the methylthioether intermediates, **1 a** and **1 b**. The aqueous phase was further extracted with portions of diethyl ether (2×25 mL) and all of the organic extracts were gathered in a round bottom flask covered by aluminum foil and immersed into an ice bath. The diethyl ether was removed under vacuum to leave the crude thioethers which were characterized by ^1^H‐NMR and found to be of a high enough purity to be used for the subsequent synthesis steps.

1‐methylthio‐2‐methoxy ethane **1 a**: ^1^H‐NMR (300 MHz, CDCl_3_): *δ*=3.49 (s, 2 H), 3.29 (s, 3 H), 2.50 (d, *J*=62.8 Hz, 2 H), 2.06 ppm (s, 3 H).

1‐methylthio‐2‐(2‐methoxyethoxy)ethane **1 b**: ^1^H‐NMR (300 MHz, CDCl_3_): *δ*=3.64–3.45 (m, 6 H), 3.31 (s, 3 H), 2.62 (t, *J=*6.9 Hz, 2 H), 2.07 ppm (s, 3 H).

**Synthesis of Dialkylether sulfonium‐based ILs [S_1,1,G1_][NTf_2_], [S_1,1,G2_][NTf_2_] and [S_1,2,G1_][NTf_2_]**: Iodomethane (107.215 g, 0.748 mol, 1.5 equiv.) was added slowly, dropwise over a period of 2 h, to the thioether (0.49 mol, 1.0 equiv.) in ultrapure water (100 mL), and the biphasic mixture was stirred at room temperature for 15 h. After this, the resulting aqueous solution was washed successively with 100 mL fractions of ethyl acetate (three times) and diethyl ether (three times) to remove excess iodomethane and traces of thioether. The resulting sulfonium iodide solution was then stirred with a solution of Li[NTf_2_] (153.805 g, 0.525 mol, 1.05 equiv.) in 150 mL of ultrapure water for 15 h at room temperature in a flask covered by aluminum foil. Thereafter, 100 mL of dichloromethane was added to dissolve the IL, which was then washed 15 times with 20 mL fractions of ultrapure water. The absence of iodide traces was verified with a silver nitrate test. Dichloromethane was then removed on a rotary evaporator and the IL was further dried under vacuum (10^−3^ mbar) at 353.15 K during three days.

**Synthesis of Dialkylether sulfonium‐based IL [S_1,2,G1_][NTf_2_]**: Diethyl sulfate (77.09 g, 0.5 mol, 1.0 equiv.) was added slowly, dropwise over a period of 2 h, to the thioether **1 a** (0.49 mol, 1.0 equiv.) in a solution of ethyl acetate (200 mL), and the mixture was stirred at room temperature for 15 h. After this, a viscous lower layer consisting of the sulfonium ethylsulfate, [S_1,2,G1_][EtSO_4_], was removed. The resulting ethyl sulfate IL was dissolved in 100 mL of ultrapure water, before being washed successively with 100 mL fractions of ethyl acetate (3 times) and diethyl ether (3 times) to remove traces of thioether. The resulting ethyl sulfate IL was subjected to metathesis with Li[NTf_2_] in an identical procedure to that described above.

**Synthesis of the Thiodiethers 2 a and 2 b**: Sodium sulfide nonahydrate (82.459 g, 0.336 mol, 1 equiv.) was dissolved in 100 mL ultrapure water. The flask was covered by an aluminum foil and immersed into an ice bath, before 98.461 g of bromoether (0.673 mol, 2 equiv.) was added dropwise over a period of 2 h. The mixture was then stirred and allowed to return slowly to room temperature for 48 h, yielding two phases. The organic phase was removed and the aqueous phase was extracted with 50 mL fractions of diethyl ether (three times) and the combined organic phase and extracts were gathered in a round‐bottom flask. The diethyl ether was removed under vacuum to leave the crude thioethers **2 a** and **2 b**. These were deemed pure enough by ^1^H‐NMR to continue with the synthesis.

Bis(2‐methoxyethyl)sulfide **2 a**: ^1^H NMR (300 MHz, CDCl_3_): *δ*=3.57 (t, *J=*6.6 Hz, 4 H), 3.37 (s, 6 H), 2.76 ppm (t, *J=*6.6 Hz, 4 H).

Bis(2‐(2‐methoxyethoxy)ethyl) sulfide **2 b**: ^1^H NMR (300 MHz, CDCl_3_): *δ*=3.69–3.59 (m, 8 H), 3.54 (s,4 H), 3.39 (s, 6 H), 2.77 ppm (t, *J=*7.0 Hz, 4 H)

**Synthesis of Alkyldiether Sulfonium‐based ILs [S_1,G1,G1_][NTf_2_] and [S_1,G2,G2_][NTf_2_]**: Iodomethane (72.420 g, 0.505 mol, 1.5 equiv.) was added slowly, dropwise over a period of 2 h, to the respective thioether (0.49 mol, 1.0 equiv.) in ultrapure water (100 mL), and the biphasic mixture was stirred at room temperature for 15 h. After this, the resulting aqueous solution was washed successively with 100 mL fractions of ethyl acetate (three times) and diethyl ether (three times) to remove excess iodomethane and traces of thioether. The resulting sulfonium iodide solution was then stirred with a solution of Li[NTf_2_] (103.534 g, 0.353 mol, 1.05 equiv.) in 150 mL of ultrapure water for 15 h at room temperature in a flask covered by aluminum foil. Thereafter, 100 mL of dichloromethane was added to dissolve the IL, which is then washed 15 times with 20 mL fractions of ultrapure water. The absence of iodide traces was verified with a silver nitrate test. Dichloromethane was then removed on a rotary evaporator.

### Chemical Characterization of the Sulfonium‐based ILs

^1^H‐ and ^13^C‐NMR spectra were recorded at 293.15 K on a Bruker Avance DPX spectrometer at 300 and 75 MHz, respectively, and are reported as Figures S1 to S5 of the SI. To avoid further contamination with moisture from the atmosphere, the ILs were stored in a glovebox under an Ar atmosphere with a moisture content below 3 ppm. Microanalysis and lithium content were performed by Analytical Services at Queen's University, Belfast.

**Dimethyl(2‐methoxyethyl)sulfonium bis{(trifluoromethyl)‐sulfonyl}imide [S_1,1,G1_][NTf_2_]**: Following the synthesis procedure, the title compound was obtained as a colorless liquid, 81 % yield. ^1^H‐NMR (300 MHz, [D_6_]DMSO): *δ*=2.88 (s, 6 H), 3.31 (s, 3 H), 3.53 (t, *J*=5.5 Hz, 2 H), 3.76 ppm (t, *J*=5.7 Hz, 2 H). ^13^C‐NMR (75 MHz, [D_6_]DMSO): *δ*=24.90, 42.75, 58.32, 65.70 (cation); 119.64 ppm (q, J_CF_ 320.25 Hz) (anion). CHNS calcd: C, 20.95; H, 3.26; N, 3.49; S, 23.97; Found: C, 21.00; H, 3.26; N, 3.37; S, 24.00. H_2_O content: 16 ppm. Li content: 16 ppm.

**Dimethyl[2‐(2‐methoxyethoxy)ethyl]sulfonium bis‐{(trifluoromethyl)sulfonyl}imide [S_1,1,G2_][NTf_2_]**: Following the synthesis procedure, the title compound was obtained as a colorless liquid, 78 % yield. ^1^H‐NMR (300 MHz, [D_6_]DMSO): *δ*=2.89 (s, 6 H), 3.26 (s, 3 H), 3.46 (m, 2 H), 3.54 (t, *J*=5.1 Hz, 2 H), 3.60 (m, 2 H), 3.86 ppm (t, *J*=5.1 Hz, 2 H). ^13^C‐NMR (75 MHz, [D_6_]DMSO): *δ*=24.80, 42.83, 58.03, 64.30, 69.50, 71.00 (cation); 119.61 ppm (q, J_CF_ 318.75 Hz) (anion). CHNS calcd: C, 24.27; H, 3.85; N, 3.14; S, 21.60; Found: C, 25.38; H, 3.80; N, 3.16; S, 22.61. H_2_O content: 26 ppm. Li content: 13 ppm.

**Ethylmethyl(2‐methoxyethyl)sulfonium bis{(trifluoro‐methyl)sulfonyl}imide [S_1,2,G1_][NTf_2_]**: Following the synthesis procedure, the title compound was obtained as a colorless liquid, 87 % yield. ^1^H‐NMR (300 MHz, [D_6_]DMSO): *δ*=1.34 (t, *J*=7.4 Hz, 3 H), 2.88 (s, 3 H), 3.23–3.44 (s+m, 5 H), 3.56 (m, 2 H), 3.77 ppm (t, *J*=5.4 Hz, 2 H). ^13^C‐NMR (75 MHz, [D_6_]DMSO): *δ*=8.25, 21.72, 35.52, 40.53, 58.26, 65.65 (cation); 119.51 ppm (q, *J*_CF_ 320.25 Hz) (anion). CHNS calcd: C, 23.13; H, 3.64; N, 3.37; S, 23.16; Found: C, 22.90; H, 3.50; N, 3.93; S, 23.08. H_2_O content: 28 ppm. Li content: 18 ppm.

**Di(2‐methoxyethyl)methylsulfonium bis{(trifluoromethyl)‐sulfonyl}imide [S_1,G1,G1_][NTf_2_]**: Following the synthesis procedure, the title compound was obtained as a colorless liquid, 68 % yield. ^1^H‐NMR (300 MHz, d_4_‐methanol): *δ*=3.00 (s, 3 H), 3.44 (s,6 H), 3.57 (t, *J*=5.7 Hz, 2 H), 3.70 ppm (m, 2 H), 3.89 ppm (t, *J*=5.3 Hz, 4 H). ^13^C‐NMR (75 MHz, d_4_‐methanol): *δ*=25.44, 33.06, 44.60, 59.80, 68.00, 73.92 (cation); 121.64 ppm (q, J_CF_ 318.75 Hz) (anion). CHNS calcd: C, 24.27; H, 3.85; N, 3.14; S, 21.60; Found: C, 24.58; H, 3.79; N, 3.37; S, 21.75. H_2_O content: 8 ppm. Li content: 87 ppm.

**Di[2‐(2‐Methoxyethoxy)ethyl]methyl sulfonium bis‐{(trifluoromethyl)sulfonyl}imide [S_1,G2,G2_][NTf_2_]**: Following the synthesis procedure, the title compound was obtained as a colorless liquid, 63 % yield. ^1^H‐NMR (300 MHz, d_4_‐methanol): *δ*=3.04 (s, 3 H), 3.40 (s, 6 H), 3.59 (m, 6 H), 3.70 (m, 6 H), 4.00 ppm (t, *J*=5.2 Hz, 4 H). ^13^C‐NMR (75 MHz, d_4_‐methanol): *δ*=25.40, 44.63, 59.51, 66.71, 71.81, 73.12 (cation); 121.66 ppm (JCF 318.75 Hz), (anion). CHNS calcd: C, 29.27; H, 4.72; N, 2.63; S, 18.03; Found: C, 29.37; H, 4.78; N, 2.38; S, 18.30. H_2_O content: 17 ppm. Li content: 10 ppm.

## Supporting information

As a service to our authors and readers, this journal provides supporting information supplied by the authors. Such materials are peer reviewed and may be re‐organized for online delivery, but are not copy‐edited or typeset. Technical support issues arising from supporting information (other than missing files) should be addressed to the authors.

Supplementary
