# Supplementary material for: Synthesis and Thermophysical Properties of Ether‐Functionalized Sulfonium Ionic Liquids as Potential Electrolytes for Electrochemical Applications
Source: Chemphyschem. 2016 Oct 27;17(23):3992–4002. doi: 10.1002/cphc.201600882 (PMC5157774; doi:10.1002/cphc.201600882)
Supplement: Supplementary file 1 — Supplementary [file CPHC-17-3992-s001.pdf]

CHEMPHYSCHEM

Supporting Information

Synthesis and Thermophysical Properties of Ether-Functionalized Sulfonium Ionic Liquids as Potential Electrolytes for Electrochemical Applications

Erwan Coadou,*[a, b] Peter Goodrich,[a] Alex R. Neale,[a] Laure Timperman,[b] Christopher Hardacre,[a, c] Johan Jacquemin,*[a, b] and Mérièm Anouti[b]

cphc_201600882_sm_miscellaneous_information.pdf

Figure S1. ^{1}H- and ^{13}C-NMR of $[S_{1,1,G1}][NTf_2]$ in d_6-DMSO.

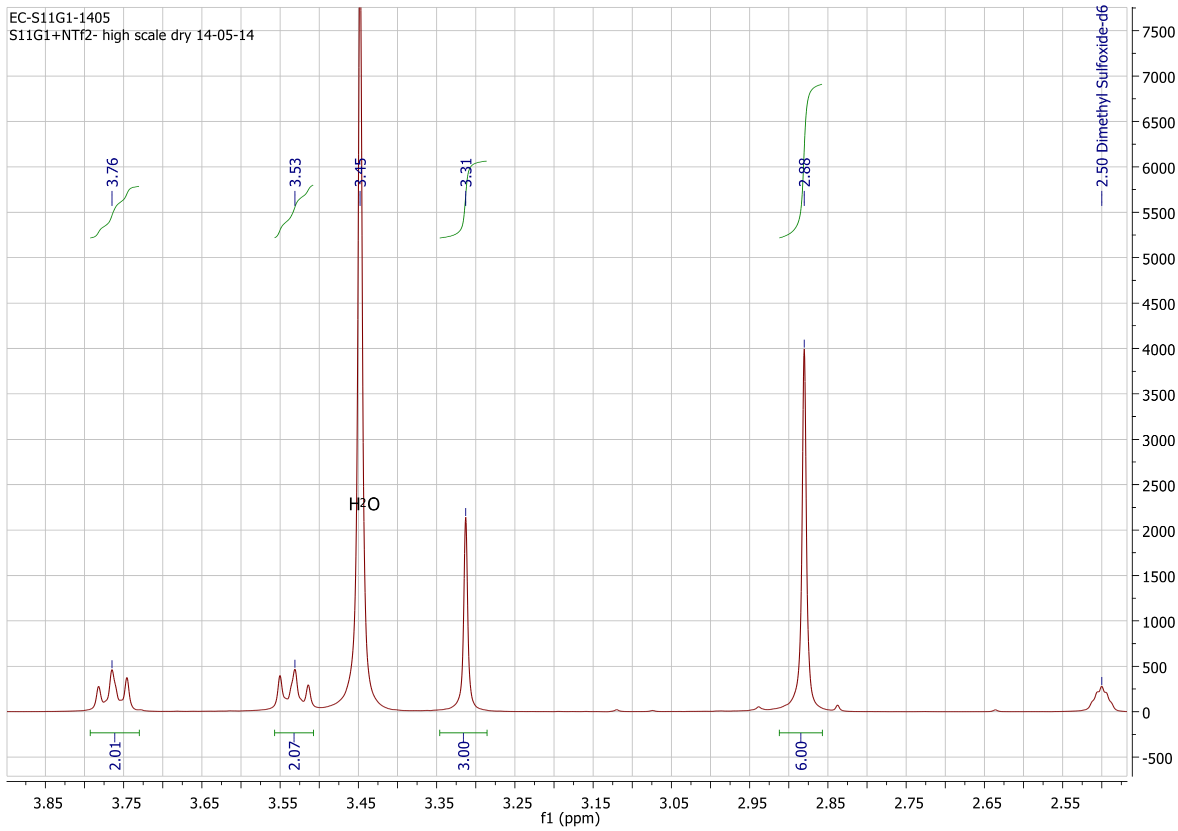

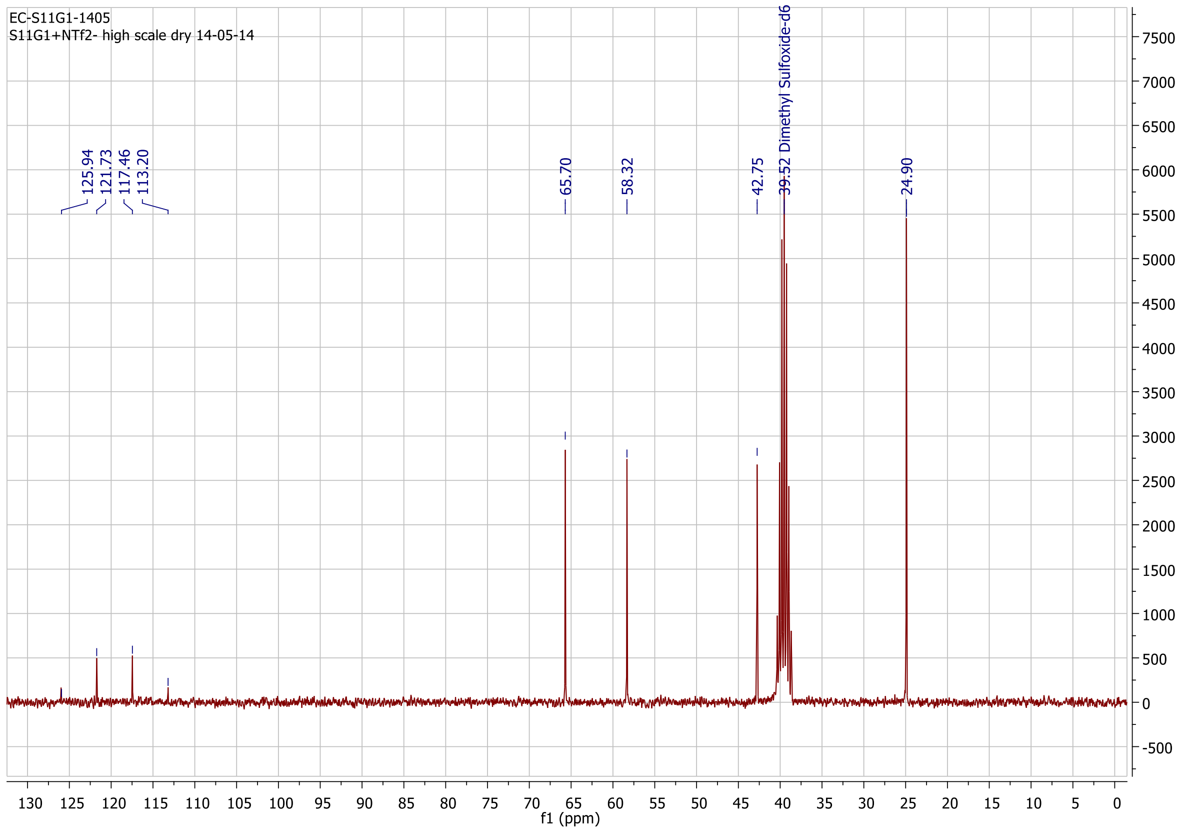

Figure S2. ^{1}H- and ^{13}C-NMR of $[S_{1,1,G2}][NTf_2]$ in d_6-DMSO.

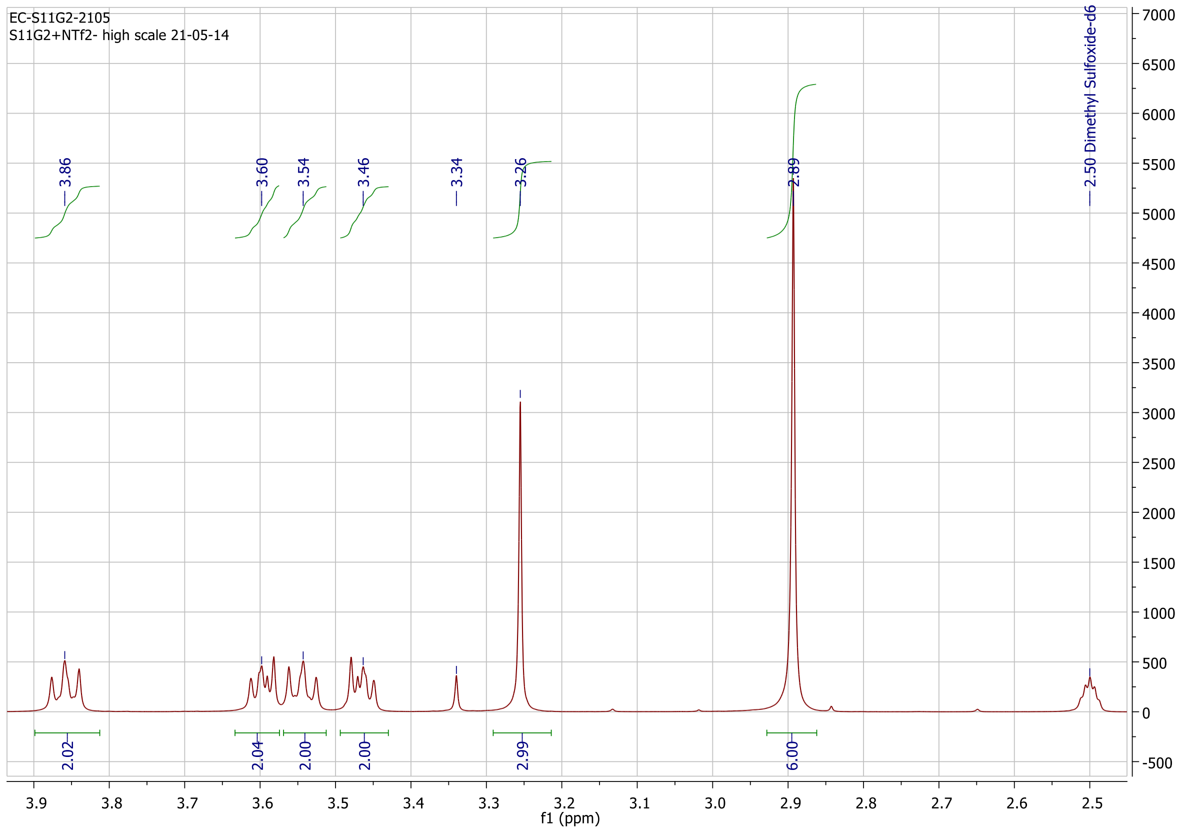

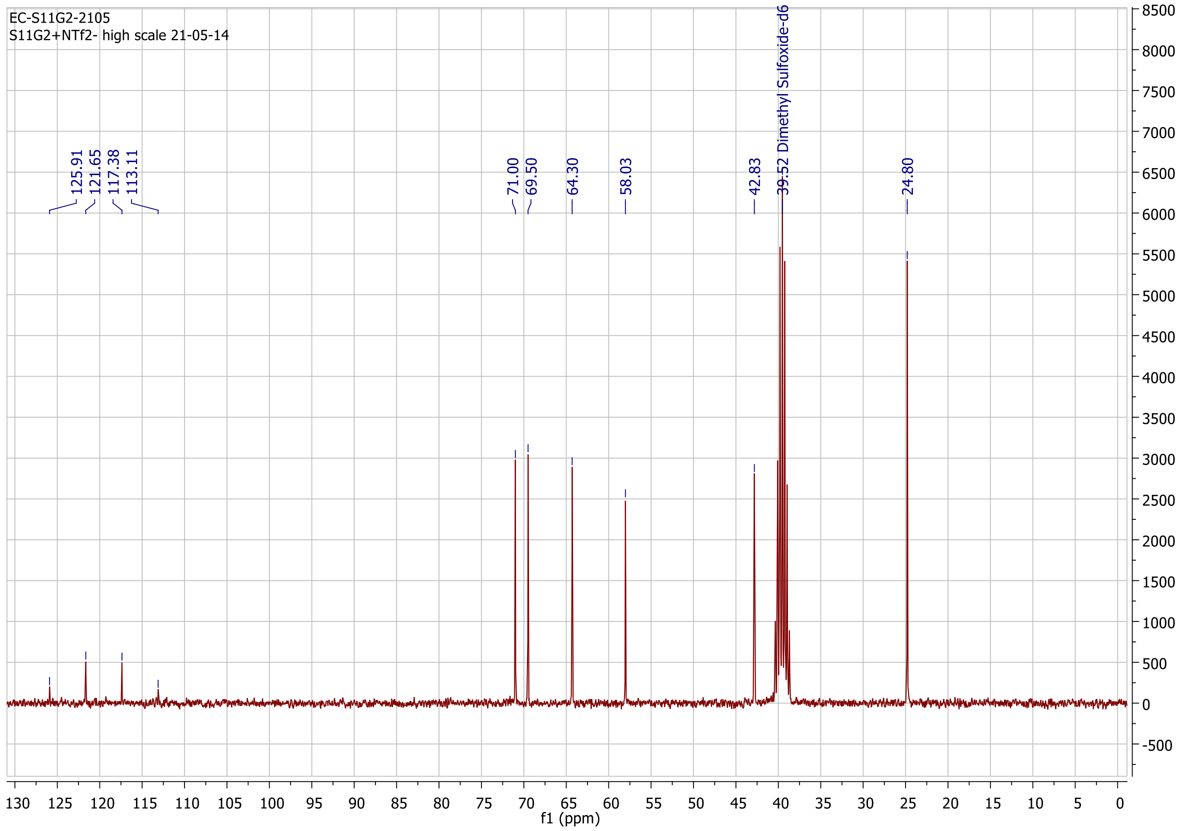

Figure S3. ^{1}H- and ^{13}C-NMR of $[S_{1,2,G1}][NTf_2]$ in d_6-DMSO.

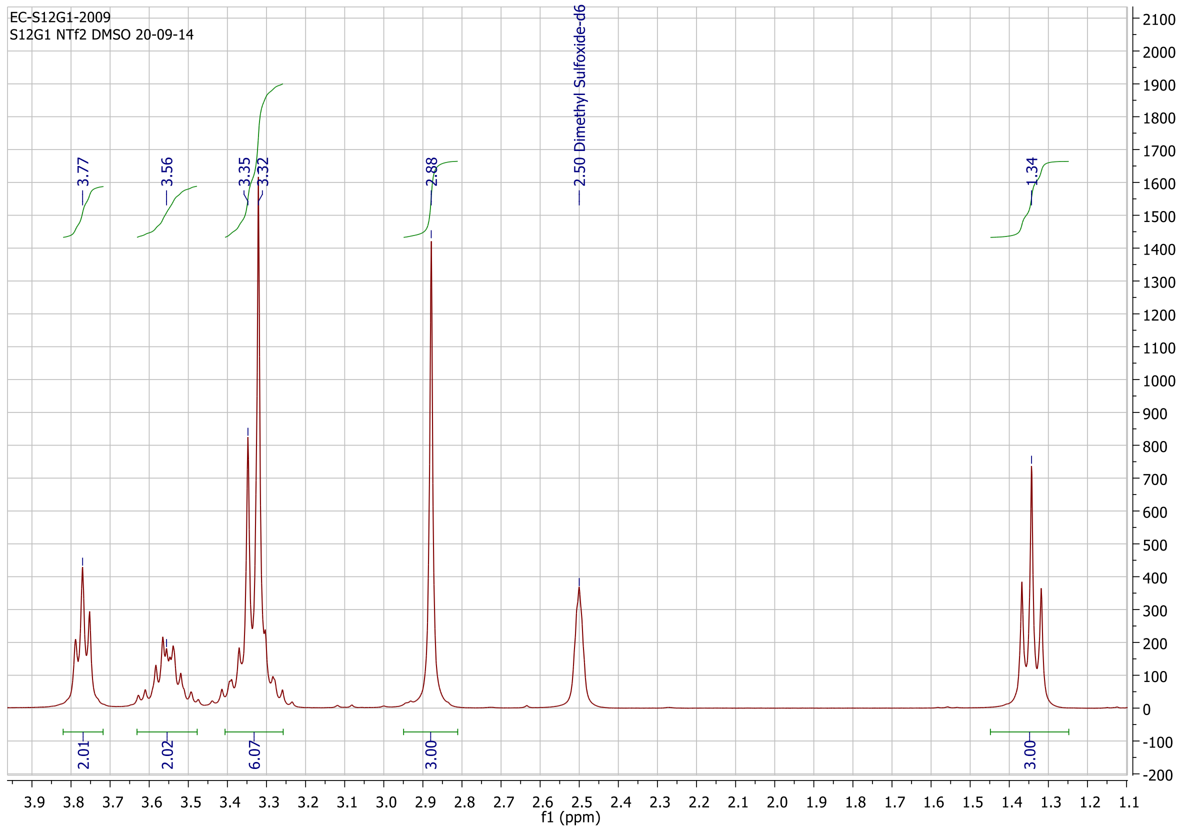

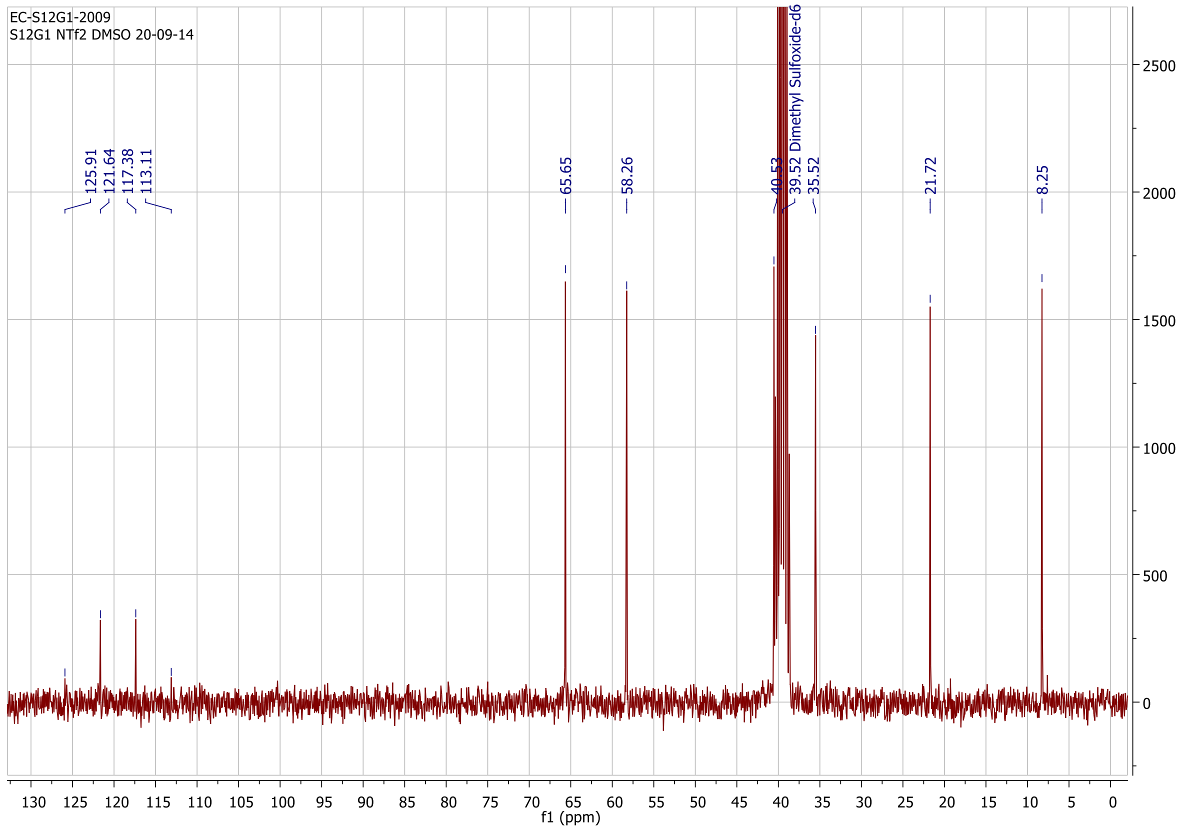

Figure S4. ^{1}H- and ^{13}C-NMR of $[S_{1,G1,G1}]$ in d_4-methanol.

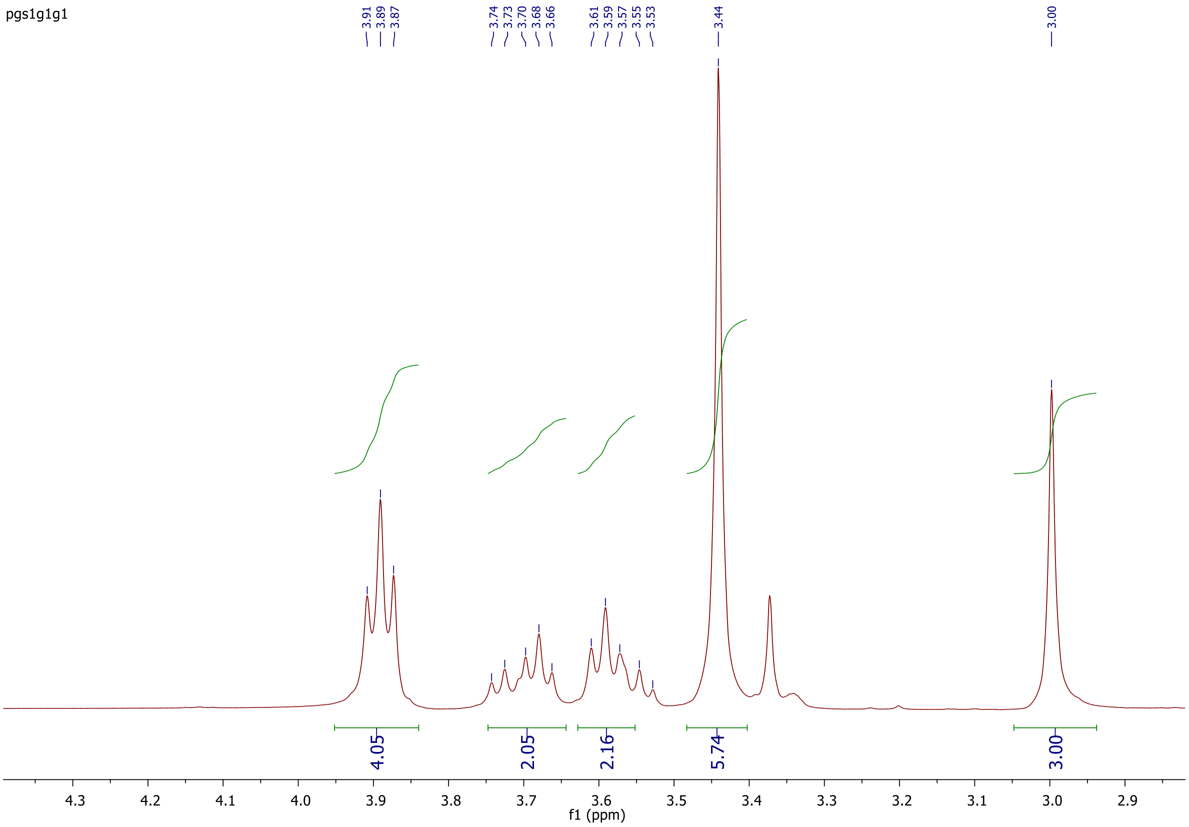

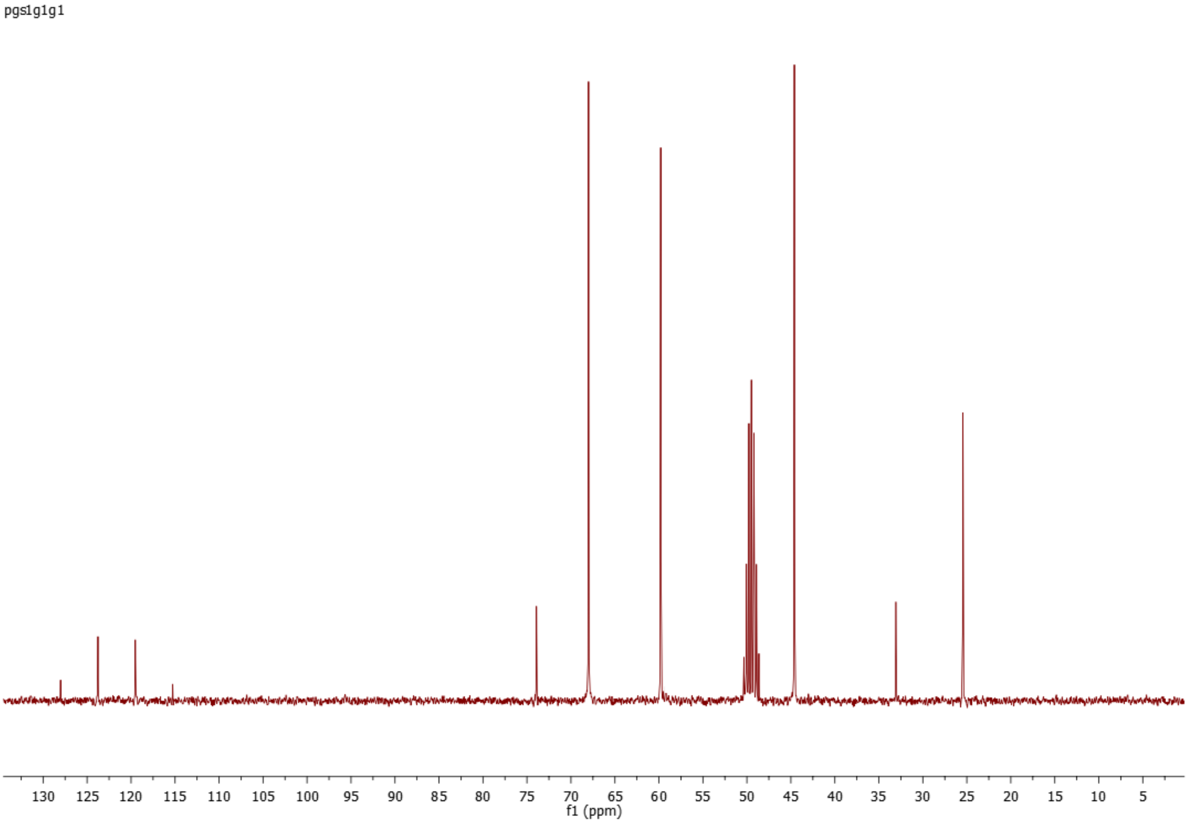

Figure S5. ^{1}H- and ^{13}C-NMR of $[S_{1,G2,G2}][NTf_2]$ in d_4-methanol.

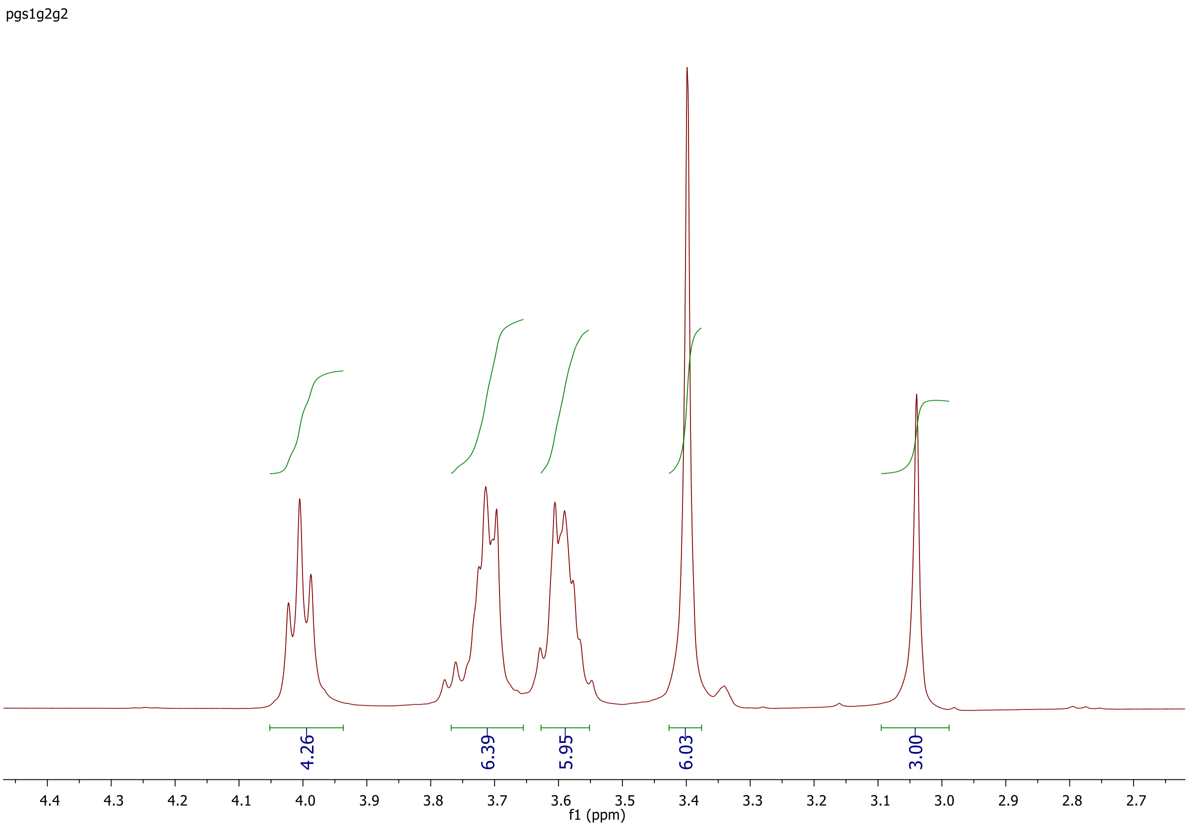

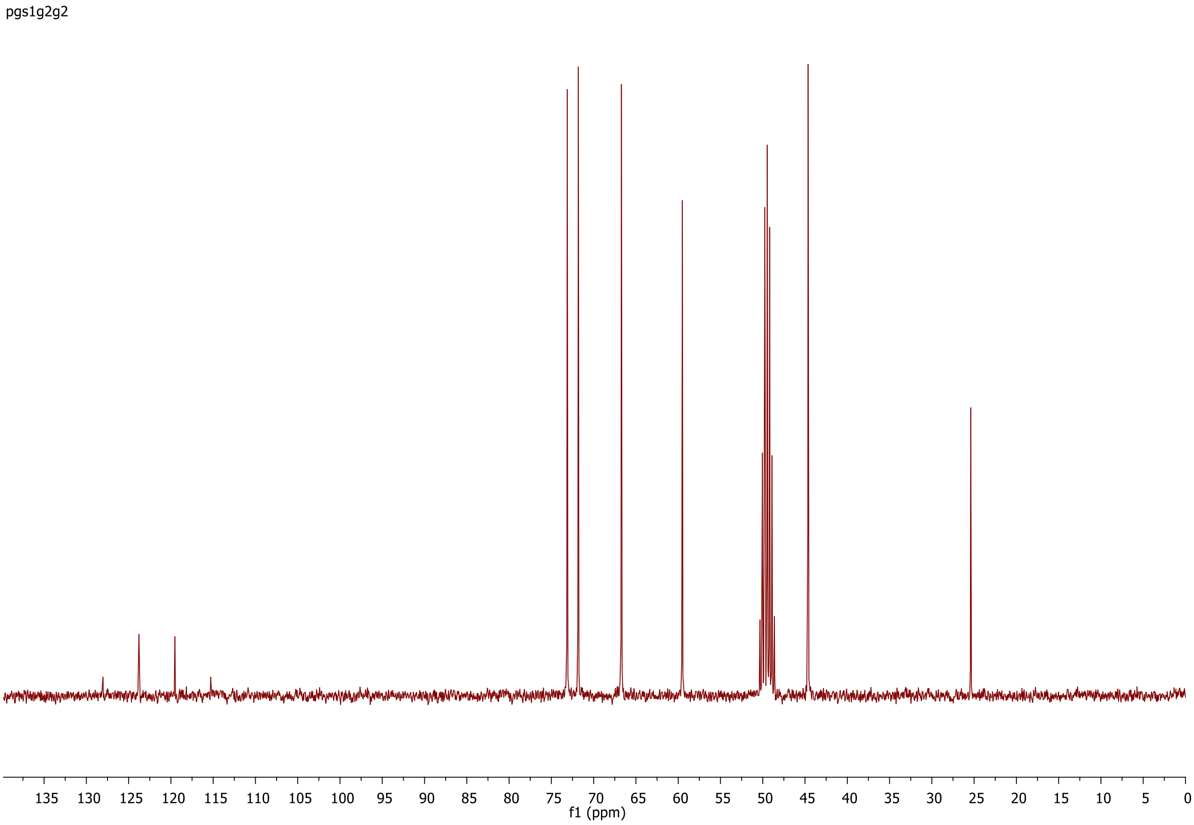

Figure S6. DSC thermograms (left) and TGA curves (right) of $[S_{1,1,G1}][NTf_2]$ (a), $[S_{1,1,G2}][NTf_2]$ (b), $[S_{1,2,G1}][NTf_2]$ (c), $[S_{1,G1,G1}][NTf_2]$ (d) and $[S_{1,G2,G2}][NTf_2]$ (e).

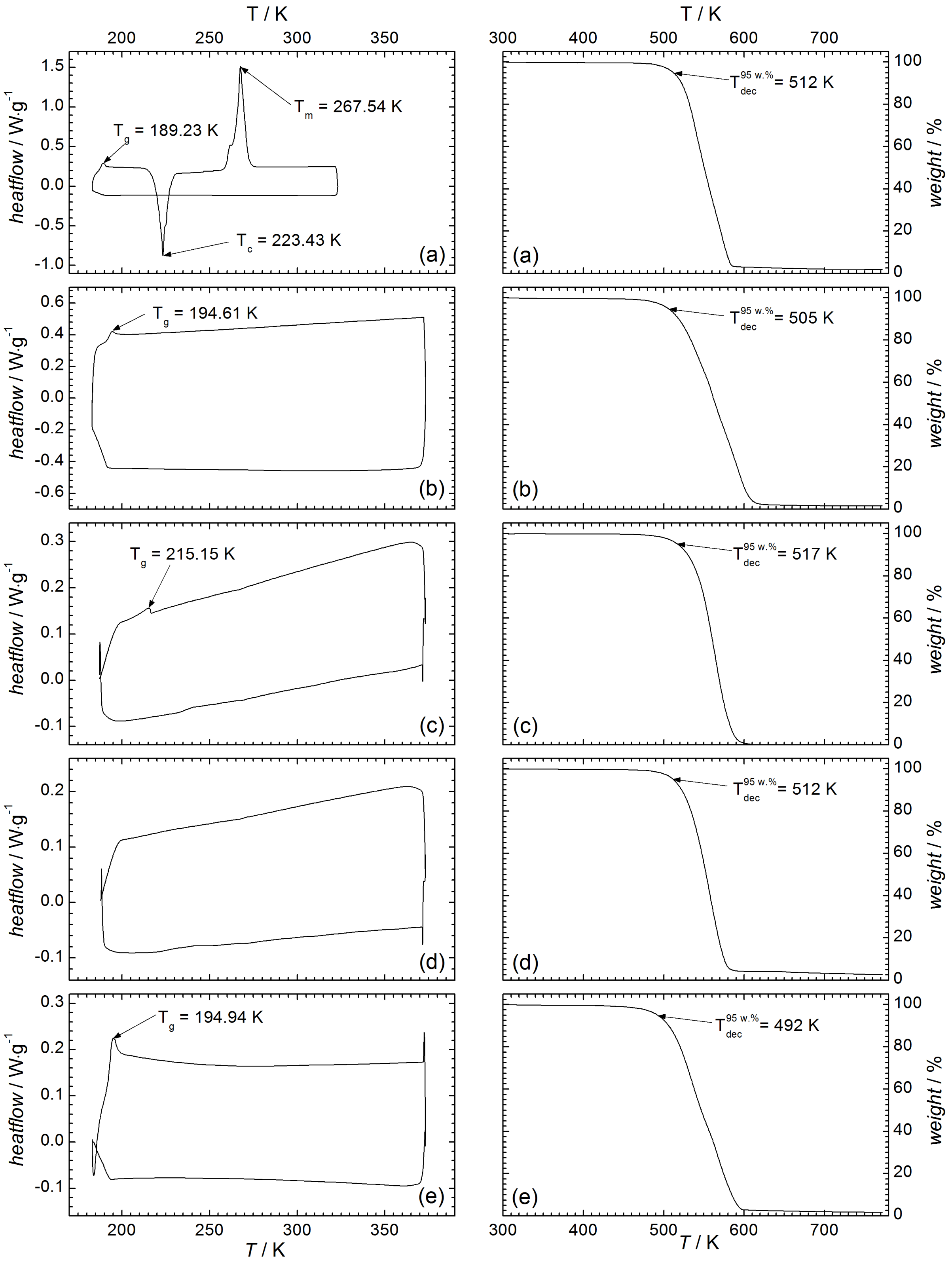

Table S1. Density data of investigated ILs as the function of the temperature from 293.15 K to 363.15 K at atmospheric pressure.

	ρ (g·cm^{-3})				
T (K)	[$S_{1,1,G1}$][NTf_2]	[$S_{1,1,G2}$][NTf_2]	[$S_{1,2,G1}$][NTf_2]	[$S_{1,G1,G1}$][NTf_2]	[$S_{1,G2,G2}$][NTf_2]
293.15	1.5280	1.4739	1.4864	1.4688	1.3928
298.15	1.5229	1.4689	1.4814	1.4638	1.3883
303.15	1.5178	1.4640	1.4764	1.4588	1.3835
313.15	1.5075	1.4540	1.4663	1.4486	1.3738
323.15	1.4975	1.4444	1.4565	1.4388	1.3644
333.15	1.4877	1.4349	1.4467	1.4290	1.3550
343.15	1.4778	1.4254	1.4371	1.4193	1.3458
353.15	1.4681	1.4160	1.4274	1.4096	1.3366
363.15	1.4585	1.4067	1.4179	1.4000	1.3274

u(ρ) = 10^{-4} g·cm^{-3}; u(T) = 0.01 K

Table S2. Fitting parameters for the density of investigated ILs.

Ionic liquid	a /g·cm^{-3}	10^{-4} · b /g·cm^{-3}·K^{-1}	R^2
[$S_{1,1,G1}$][NTf_2]	1.8191	-9.941	0.9999
[$S_{1,1,G2}$][NTf_2]	1.7551	-9.603	0.9999
[$S_{1,2,G1}$][NTf_2]	1.7733	-9.796	0.9999
[$S_{1,G1,G1}$][NTf_2]	1.7569	-9.836	0.9999
[$S_{1,G2,G2}$][NTf_2]	1.6675	-9.371	0.9999

Table S3. Viscosity data of investigated ILs as the function of the temperature from 267 K to 370 K at atmospheric pressure.

$[S_{1,1,G1}][NTf_2]$		$[S_{1,1,G2}][NTf_2]$		$[S_{1,2,G1}][NTf_2]$		$[S_{1,G1,G1}][NTf_2]$		$[S_{1,G2,G2}][NTf_2]$	
T (K)	*η* (mPa·s)	*T* (K)	*η* (mPa·s)	*T* (K)	*η* (mPa·s)	*T* (K)	*η* (mPa·s)	*T* (K)	*η* (mPa·s)
270.65	173.9	270.05	155.0	293.15	37.16	293.25	34.35	267.85	177.7
274.35	156.4	274.25	127.0	298.15	31.27	298.25	27.81	272.85	140.4
282.45	104.5	282.95	77.42	303.35	25.86	303.35	22.66	280.75	90.55
288.55	74.80	289.65	51.68	308.35	21.47	308.35	18.59	287.55	61.59
294.25	56.22	296.15	36.69	313.25	18.04	313.35	15.53	294.25	43.65
300.05	43.60	302.55	26.72	318.25	15.36	318.35	13.24	300.85	31.57
305.85	34.57	308.95	20.79	323.35	13.30	323.35	11.53	307.45	23.81
311.55	27.90	315.45	16.39	328.35	11.74	328.35	10.25	313.95	18.39
317.25	22.93	321.95	13.62	333.25	10.63	333.25	9.167	320.55	15.11
323.05	19.22	328.35	11.37	338.25	9.623	338.35	8.187	327.15	12.23
328.75	16.34	334.65	9.592	343.25	8.649	343.35	7.296	333.75	10.49
334.55	14.01	341.15	8.244	348.35	7.672	348.25	6.426	340.35	9.098
340.25	12.11	347.55	7.246	353.25	6.827	353.25	5.665	346.85	7.838
345.95	10.58	353.95	6.331	358.15	6.078	358.35	5.021	353.45	6.582
351.65	9.292	360.35	5.494	363.15	5.453	363.25	4.507	360.05	5.411
357.45	8.103	366.75	4.722	368.15	4.904	368.15	4.239	366.65	4.588

$u(\eta) = (0.01 \times \eta)$ mPa·s; $u(T) = 0.01$ K

Table S4. Conductivity data of investigated ILs as the function of the temperature from 263.15 K to 353.15 K at atmospheric pressure.

$[S_{1,1,G1}][NTf_2]$		$[S_{1,1,G2}][NTf_2]$		$[S_{1,2,G1}][NTf_2]$		$[S_{1,G1,G1}][NTf_2]$		$[S_{1,G2,G2}][NTf_2]$	
T (K)	σ (mS·cm^{-1})	T (K)	σ (mS·cm^{-1})	T (K)	σ (mS·cm^{-1})	T (K)	σ (mS·cm^{-1})	T (K)	σ (mS·cm^{-1})
294.35	3.373	294.55	2.450	263.15	0.789	265.85	0.635	267.05	0.395
298.85	4.036	298.95	2.946	265.15	0.897	268.15	0.737	268.15	0.423
303.35	4.802	303.15	3.476	268.05	1.057	273.15	0.976	273.15	0.575
308.35	5.755	308.25	4.219	273.05	1.362	278.15	1.261	278.15	0.761
313.15	6.682	313.35	5.007	278.25	2.200	283.05	2.050	283.15	0.974
318.25	7.837	318.15	5.880	282.95	2.740	288.15	2.600	288.15	1.556
323.35	9.099	322.55	6.711	288.05	3.410	293.15	3.240	293.15	1.943
328.45	10.44	328.25	7.850	293.15	4.190	298.15	3.950	298.15	2.390
333.35	11.86	332.85	8.877	298.15	5.030	303.15	4.740	303.15	2.900
338.15	13.30	338.05	10.10	302.95	5.920	308.05	5.620	308.15	3.470
344.05	15.25	343.25	11.43	308.05	6.960	313.15	6.600	313.15	4.090
348.45	16.77	348.25	12.73	313.15	8.070	318.15	7.620	318.15	4.760
352.85	18.32	353.25	14.10	318.05	9.230	323.15	8.740	323.15	5.480
				323.15	10.52	328.15	9.930	328.15	6.240
				328.15	11.86	333.15	11.19	333.15	7.060
				333.05	13.24	338.15	12.50	338.15	7.930
				338.15	14.77	343.15	13.90	343.15	8.860
				343.15	16.79	348.15	15.79	348.15	9.810
				348.05	18.48	353.05	17.34	353.15	10.81
				353.05	20.20				

u(σ) = (0.01 × σ) mS·cm^{-1}; u(T) = 0.05 K

Table S5. Arrhenius and VTF fitting parameters for the conductivity and viscosity of investigated ILs.

Ionic liquid	Arrhenius			VTF			
	σ_0 (10^3·mS·cm^{-1})	E_a^{σ} (kJ·mol^{-1})	R^2	σ_0 (mS·cm^{-1})	B^{σ} (K)	T_0 (K)	R^2
$[S_{1,1,G1}][NTf_2]$	60.861	23.734	0.9982	854.43	734.81	161.57	0.9999
$[S_{1,1,G2}][NTf_2]$	57.003	24.327	0.9974	521.81	647.98	173.77	0.9999
$[S_{1,2,G1}][NTf_2]$	48.692	22.774	0.9957	613.24	642.32	165.00	0.9996
$[S_{1,G1,G1}][NTf_2]$	60.924	23.891	0.9951	527.72	619.09	172.19	0.9994
$[S_{1,G2,G2}][NTf_2]$	45.958	24.419	0.9929	205.08	475.26	191.86	0.9997
Ionic liquid	Arrhenius			VTF			
	η_0 (10^{-3}·mPa·s)	E_a^{η} (kJ·mol^{-1})	R^2	η_0 (10^{-3} mPa·s)	B^{η} (K)	T_0 (K)	R^2
$[S_{1,1,G1}][NTf_2]$	0.177	31.123	0.9967	122.51	851.03	155.03	0.9988
$[S_{1,1,G2}][NTf_2]$	0.024	35.208	0.9976	172.23	626.32	179.04	0.9989
$[S_{1,2,G1}][NTf_2]$	1.175	25.221	0.9969	113.19	829.12	150.29	0.9987
$[S_{1,G1,G1}][NTf_2]$	0.568	26.766	0.9951	104.39	774.27	159.39	0.9988
$[S_{1,G2,G2}][NTf_2]$	0.043	33.941	0.9984	76.82	868.48	156.84	0.9987

Table S6. Walden product of investigated $[NTf_2]^-$-based sulfonium ILs as the function of temperature from 293.15 K to 353.15 K.

	$[S_{1,1,G1}]^+$	$[S_{1,1,G2}]^+$	$[S_{1,2,G1}]^+$	$[S_{1,G1,G1}]^+$	$[S_{1,G2,G2}]^+$
T /K	Walden product: $W = \Lambda \cdot \eta$ / $S \cdot cm^2 \cdot P \cdot mol^{-1}$				
293.15	0.490	0.289	0.428	0.327	0.324
303.15	0.482	0.284	0.424	0.325	0.321
313.15	0.476	0.281	0.418	0.322	0.315
323.15	0.469	0.279	0.413	0.319	0.306
333.15	0.463	0.279	0.407	0.316	0.296
343.15	0.458	0.278	0.401	0.313	0.286
353.15	0.453	0.278	0.396	0.309	0.276

Table S7. Linear fitting parameters for the fractional Walden rule (eq. 5) of selected $[NTf_2]^-$-based sulfonium ILs.

Cation	α	$\log(W' / S \cdot cm^2 \cdot mol^{-1})$	R^2
$[S_{1,1,G1}]^+$	0.9584	-0.2995	1.00000
$[S_{1,1,G2}]^+$	0.9810	-0.5350	0.99995
$[S_{1,2,G1}]^+$	0.9542	-0.3465	0.99995
$[S_{1,G1,G1}]^+$	0.9692	-0.4690	0.99996
$[S_{1,G2,G2}]^+$	0.9161	-0.4508	0.99932

Table S8. HOMO and LUMO Energies and Surfaces of Selected Ions and Ionic Liquids.

$[S_{1,1,4}]^+$				
	HOMO		LUMO	
Conformer	Surface	Energy (eV)	Surface	Energy (eV)
1	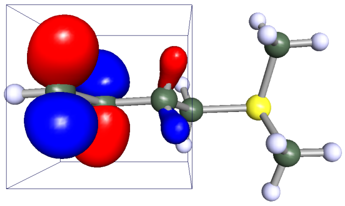	-12.547	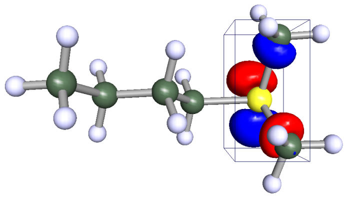	-4.528
2	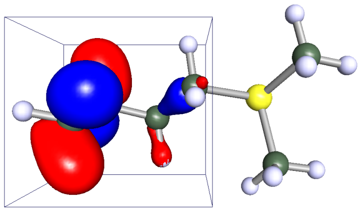	-12.564	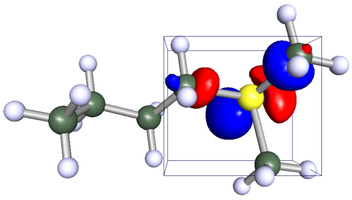	-4.631
3	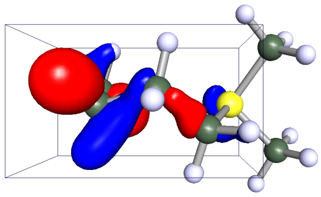	-12.591	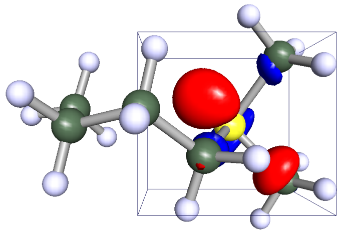	-4.642
Average		-12.567		-4.601

Table S8. *Continued...*

$[S_{1,1,G1}]^+$				
	HOMO		LUMO	
Conformer	Surface	Energy (eV)	Surface	Energy (eV)
1	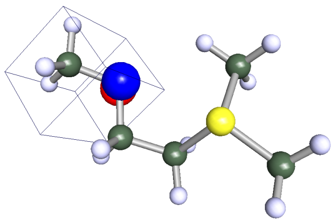	-11.162	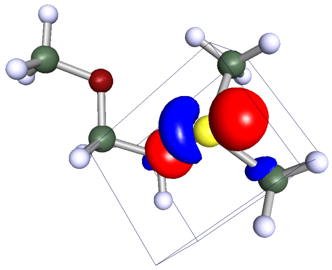	-4.585
2	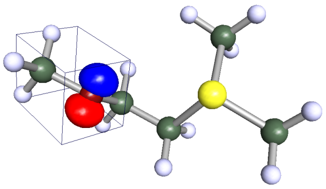	-11.083	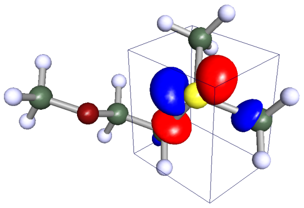	-4.544
3	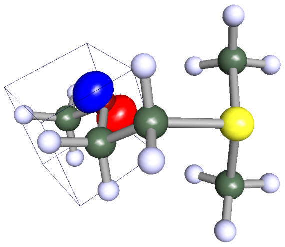	-11.344	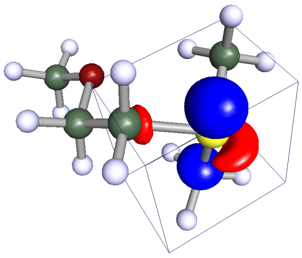	-4.580
4	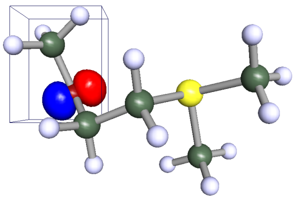	-11.214	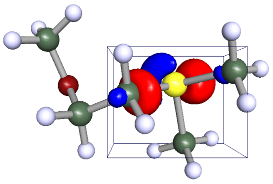	-4.618
Average		-11.201		-4.582

Table S8. *Continued...*

	$[S_{1,1,G2}]^+$			
	HOMO		LUMO	
Conformer	Surface	Energy (eV)	Surface	Energy (eV)
1	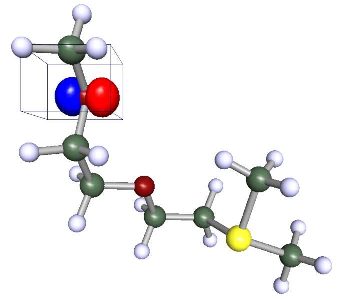	-9.709	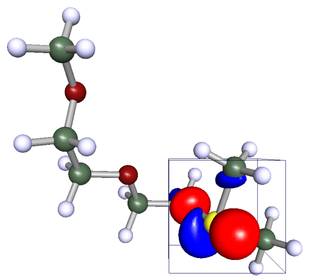	-4.487
2	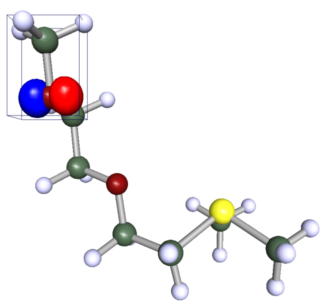	-9.543	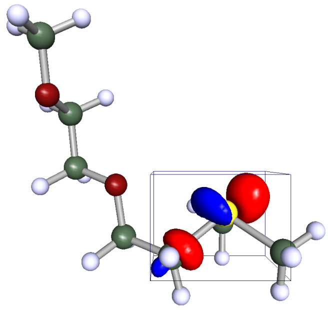	-4.455
3	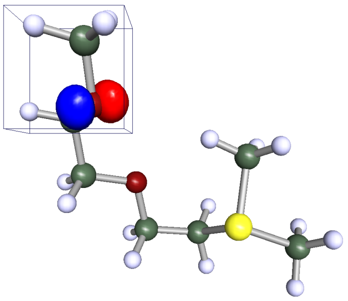	-9.886	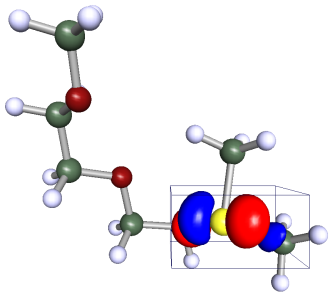	-4.433
4	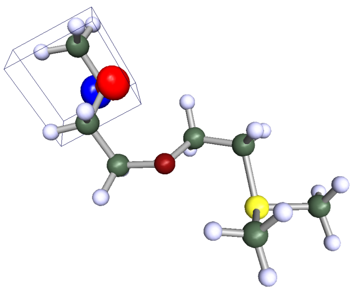	-9.864	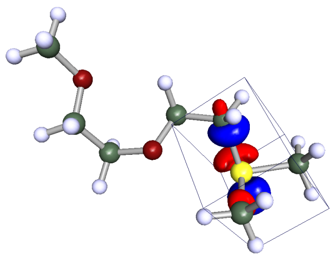	-4.392

Table S8. *Continued...*

	$[S_{1,1,G2}]^+$			
	HOMO		LUMO	
Conformer	Surface	Energy (eV)	Surface	Energy (eV)
5	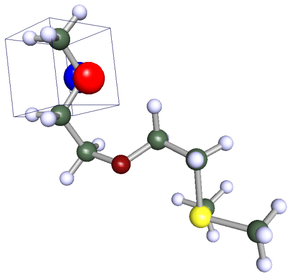	-9.851	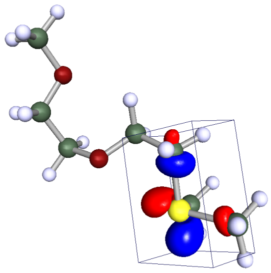	-4.351
6	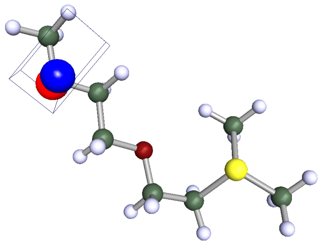	-9.652	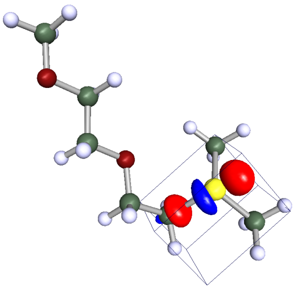	-4.572
7	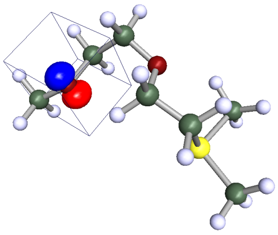	-10.071	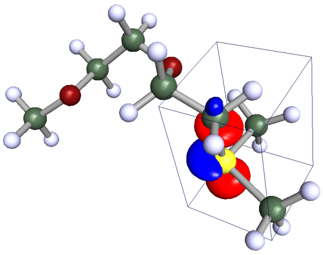	-4.362
8	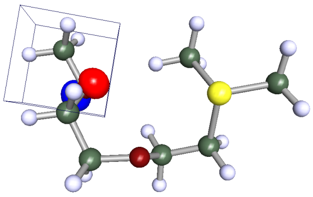	-10.444	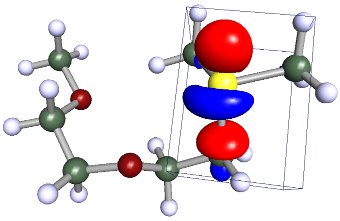	-4.275

Table S8. *Continued...*

	$[S_{1,1,G2}]^+$			
	HOMO		LUMO	
Conformer	Surface	Energy (eV)	Surface	Energy (eV)
9	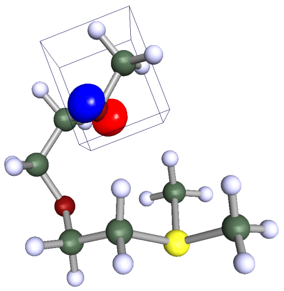	-10.683	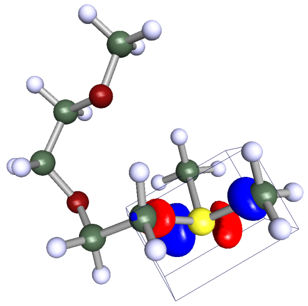	-4.408
Average		-9.967		-4.415

	$[S_{1,2,G1}]^+$			
	HOMO		LUMO	
Conformer	Surface	Energy (eV)	Surface	Energy (eV)
1	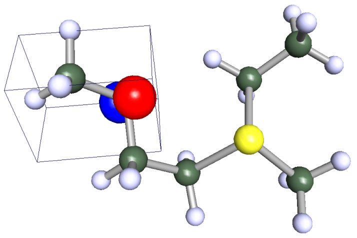	-11.083	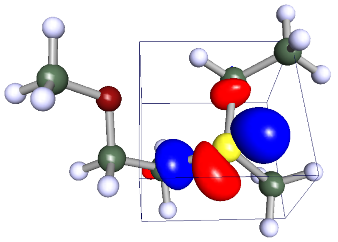	-4.607
2	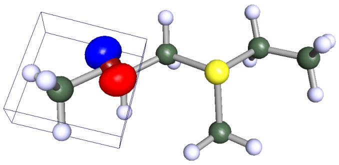	-10.985	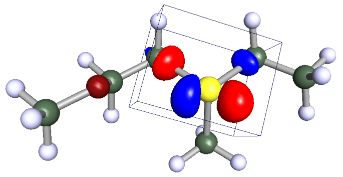	-4.620

Table S8. *Continued...*

	$[S_{1,2,G1}]^+$			
	HOMO		LUMO	
Conformer	Surface	Energy (eV)	Surface	Energy (eV)
3	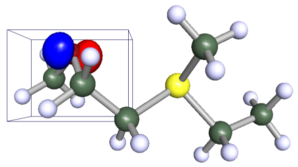	-11.100	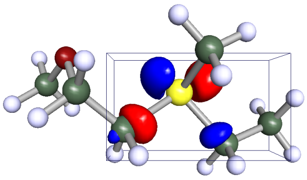	-4.550
4	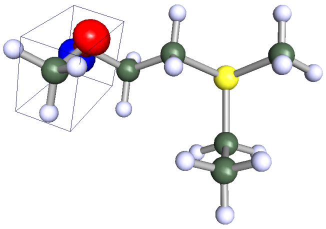	-10.955	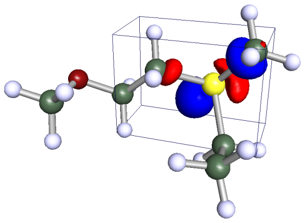	-4.634
5	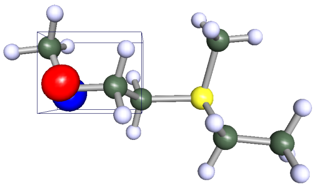	-10.988	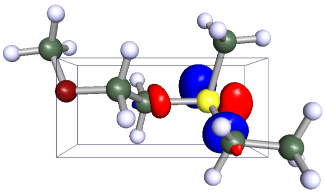	-4.601
Average		-11.022		-4.603

Table S8. *Continued...*

	$[S_{1,G1,G1}]^{+}$			
	HOMO		LUMO	
Conformer	Surface	Energy (eV)	Surface	Energy (eV)
1	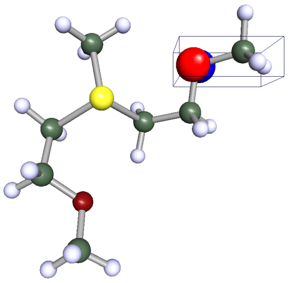	-10.890	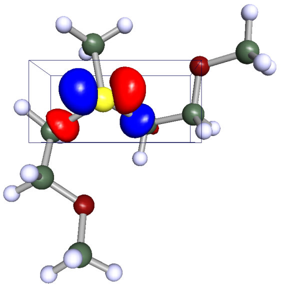	-4.283
2	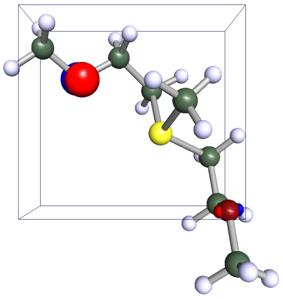	-10.901	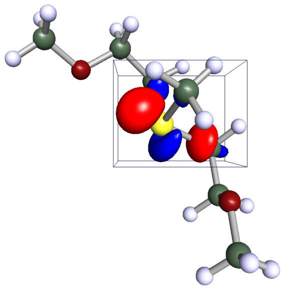	-4.250
3	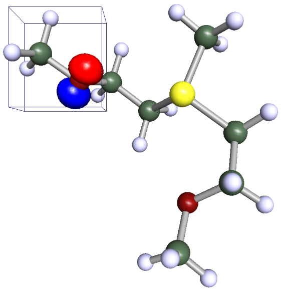	-10.806	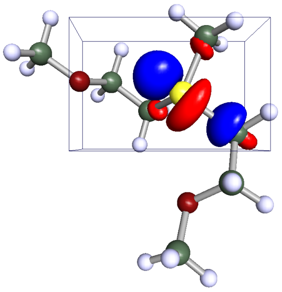	-4.275
4	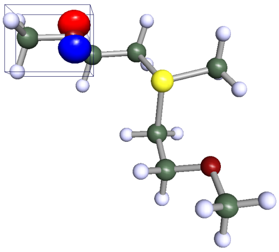	-10.849	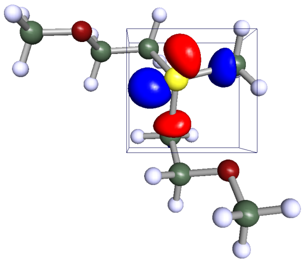	-4.308

Table S8. *Continued...*

	$[S_{1,G1,G1}]^{+}$			
	HOMO		LUMO	
Conformer	Surface	Energy (eV)	Surface	Energy (eV)
5	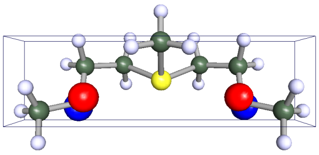	-10.936	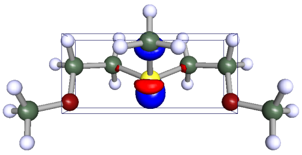	-4.142
6	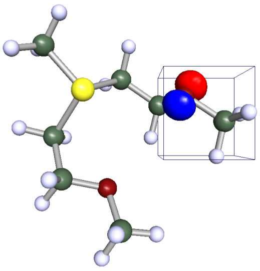	-10.563	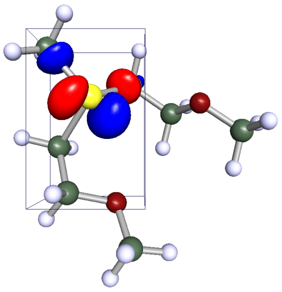	-4.460
7	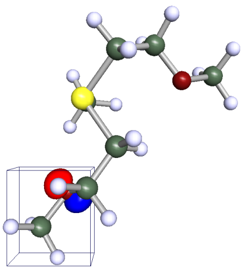	-10.882	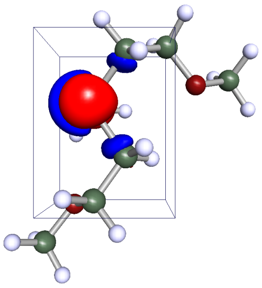	-4.267
8	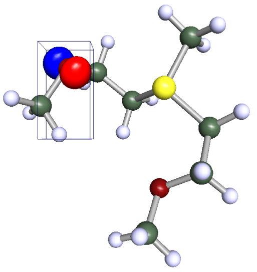	-10.871	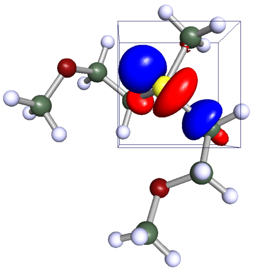	-4.359

Table S8. *Continued...*

	$[S_{1,G1,G1}]^{+}$			
	HOMO		LUMO	
Conformer	Surface	Energy (eV)	Surface	Energy (eV)
9	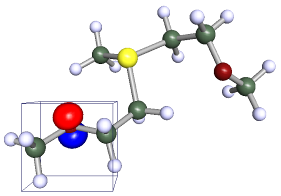	-10.936	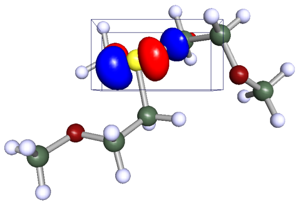	-4.389
10	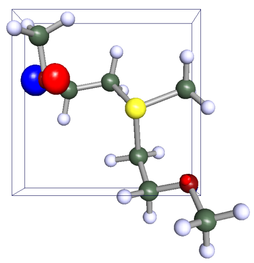	-10.953	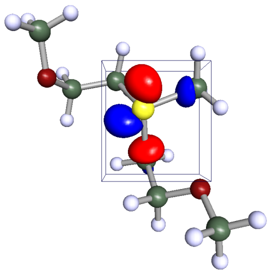	-4.386
Average		-10.859		-4.312

Table S8. *Continued...*

	$[S_{1,G2,G2}]^+$			
	HOMO		LUMO	
Conformer	Surface	Energy (eV)	Surface	Energy (eV)
1	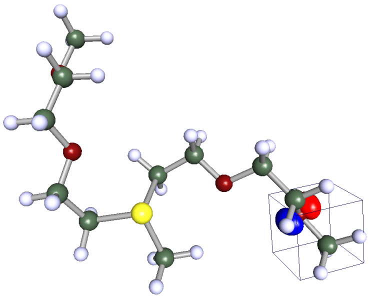	-9.559	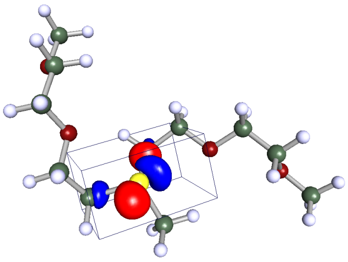	-4.125
2	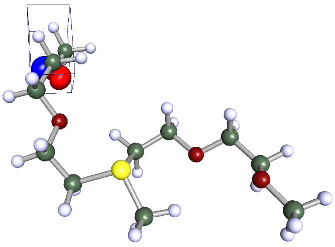	-9.649	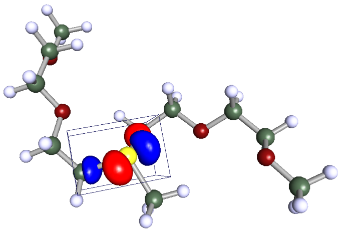	-4.087
3	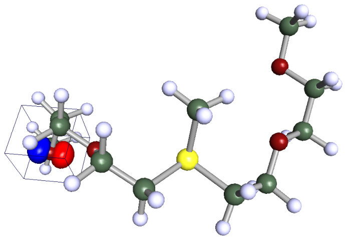	-9.374	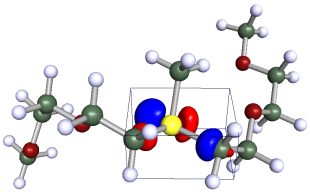	-4.060
4	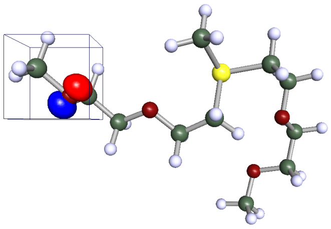	-9.494	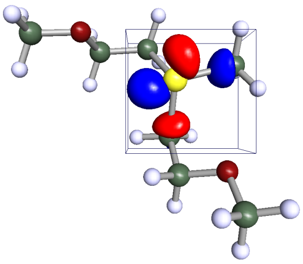	-4.052

Table S8. *Continued...*

	$[S_{1,G2,G2}]^+$			
	HOMO		LUMO	
Conformer	Surface	Energy (eV)	Surface	Energy (eV)
5	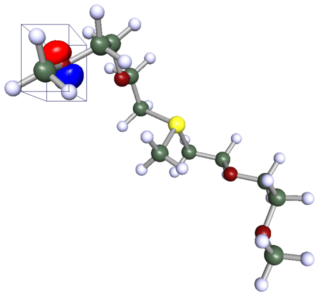	-9.554	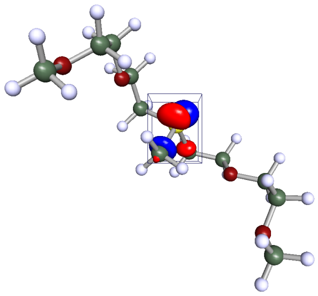	-4.095
6	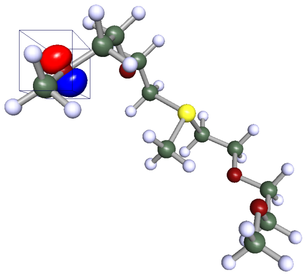	-9.521	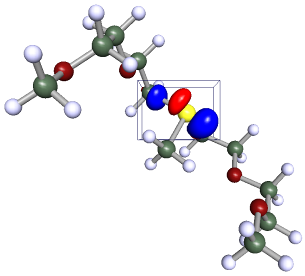	-4.041
7	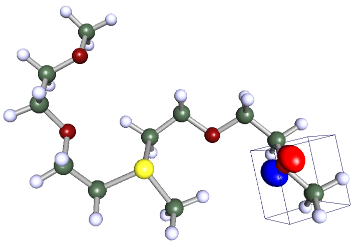	-9.668	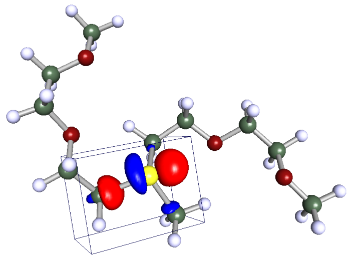	-4.011
8	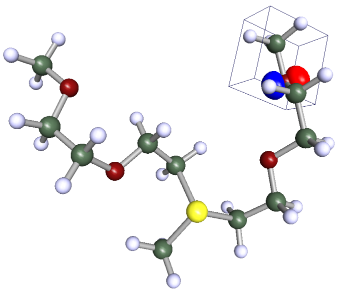	-9.608	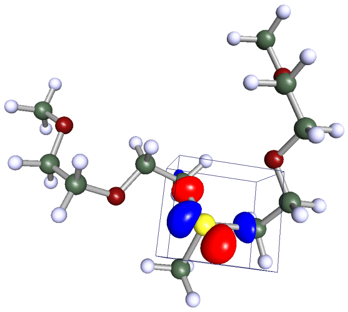	-4.041

Table S8. *Continued...*

		$[S_{1,G2,G2}]^+$		
	HOMO		LUMO	
Conformer	Surface	Energy (eV)	Surface	Energy (eV)
9	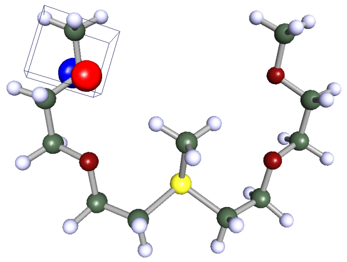	-9.641	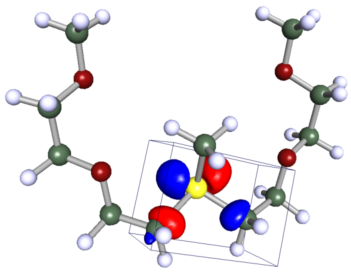	-4.035
10	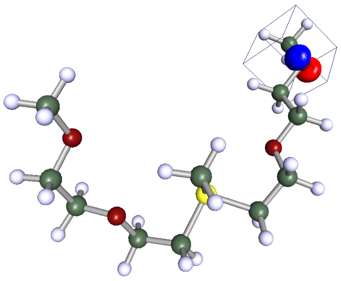	-9.396	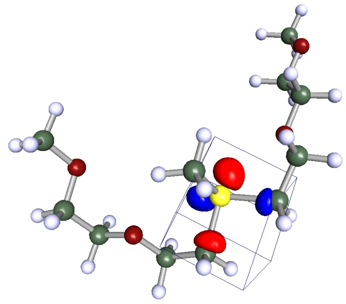	-4.120
Average		-9.547		-4.067

Table S8. *Continued...*

	[NTf$_2$]$^-$			
	HOMO		LUMO	
Conformer	Surface	Energy (eV)	Surface	Energy (eV)
trans	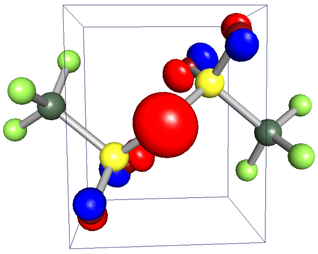	-4.188	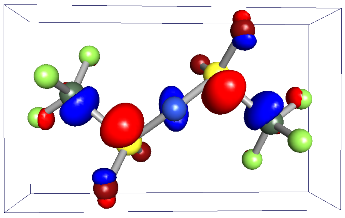	3.347
cis	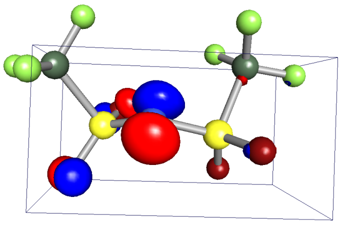	-3.967	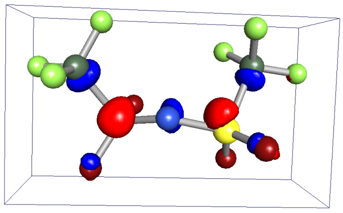	3.380
3	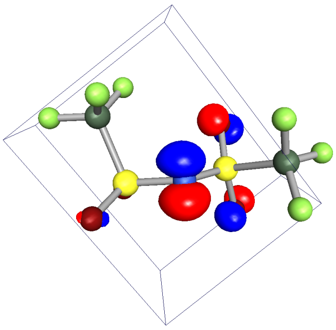	-3.889	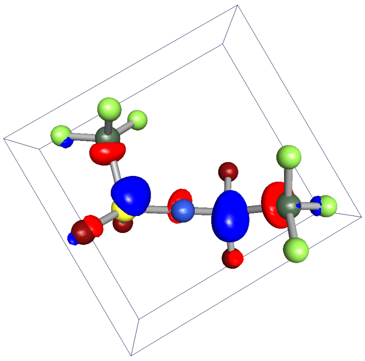	3.358
Average		-4.015		3.362

Table S8. *Continued...*

$[S_{1,1,G1}][NTf_2]$				
	HOMO		LUMO	
Conformer	Surface	Energy (eV)	Surface	Energy (eV)
trans	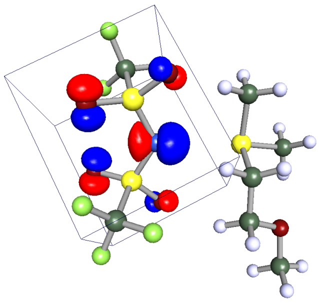	-7.538	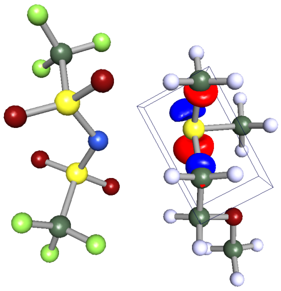	-1.143
Total energy = -2498.6574 Hartree				
cis	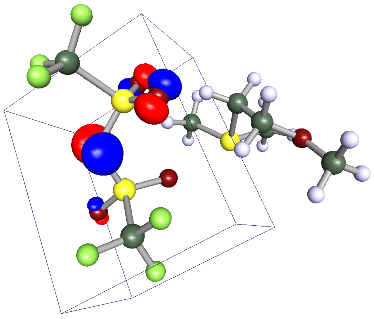	-7121	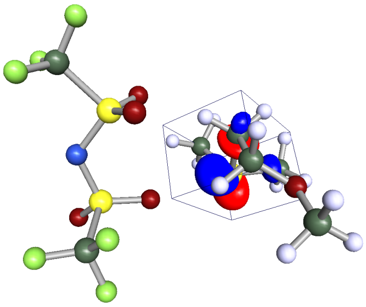	-1.007
Total energy = -2498.6597 Hartree				
